# Correlates of rate heterogeneity in avian ecomorphological traits

**DOI:** 10.1111/ele.13131

**Published:** 2018-08-21

**Authors:** A. M. Chira, C. R. Cooney, J. A. Bright, E. J. R. Capp, E. C. Hughes, C. J. A. Moody, L. O. Nouri, Z. K. Varley, G. H. Thomas

**Affiliations:** ^1^ Department of Animal and Plant Sciences University of Sheffield Sheffield S10 2TN UK; ^2^ School of Geosciences University of South Florida Tampa FL USA; ^3^ Bird Group Department of Life Sciences The Natural History Museum Tring Hertfordshire UK

**Keywords:** Ecological opportunity, morphological distinctiveness, rate heterogeneity, trait evolution

## Abstract

Heterogeneity in rates of trait evolution is widespread, but it remains unclear which processes drive fast and slow character divergence across global radiations. Here, we test multiple hypotheses for explaining rate variation in an ecomorphological trait (beak shape) across a globally distributed group (birds). We find low support that variation in evolutionary rates of species is correlated with life history, environmental mutagenic factors, range size, number of competitors, or living on islands. Indeed, after controlling for the negative effect of species' age, 80% of variation in species‐specific evolutionary rates remains unexplained. At the clade level, high evolutionary rates are associated with unusual phenotypes or high species richness. Taken together, these results imply that macroevolutionary rates of ecomorphological traits are governed by both ecological opportunity in distinct adaptive zones and niche differentiation among closely related species.

## Introduction

Phenotypic diversity accumulates via different mechanisms and at different speeds, and understanding which factors predict the tempo of phenotypic diversification represents a longstanding question in evolutionary biology (Simpson 1953; Pagel 1999). Candidate drivers include predictors related to a general increase in the potential for genetic variability and fixation rates (mostly associated with rates of molecular evolution), but also predictors relevant only for specific types of traits, for example diet links with the rate of jaw morphology evolution in *Centrachidae* (Collar *et al*. 2009). Rates of ecomorphological trait evolution in particular have received considerable interest. The classic research on Darwin's finches in the Galapagos has shown that fluctuations in resource availability, colonisation of islands and interspecific competition can cause exceptionally rapid differentiation in beak size (Grant & Grant 2003, 2006). Recently, studies addressed patterns of trait evolution for entire global radiations, involving thousands of species (Venditti *et al*. 2011; Rabosky *et al*. 2013; Cooney *et al*. 2017), but whether it is possible to identify the factors that accelerate or constrain phenotypic evolution in ecological traits at broad taxonomic scales remains unclear.

The pace of evolution depends in part on factors that increase the potential for genetic variability in populations (Simpson 1953). Aspects of species life history, such as faster turnover of generations and increased levels of fecundity increase the potential for copy error (Bromham 2009; Lanfear *et al*. 2010b; Bromham 2011; Bromham *et al*. 2015). Similarly, species with shorter life spans, smaller body sizes and higher metabolic rates suffer from a less efficient DNA repair process (Galtier *et al*. 2009). An increase in the total number of gene changes can be an important source of variation for selection to act on, and also a rapid turnover of generations should speed up the process of fixation under selection. However, the evidence that species life histories are linked with the rate of molecular evolution is mixed (Mooers & Harvey 1994; Smith & Donoghue 2008; Lanfear *et al*. 2010a; Thomas *et al*. 2010; Thomson *et al*. 2014). The potential for genetic variability has also been linked to factors extrinsic to species. Specifically, it has been hypothesised that abiotic mutagenic factors such as increased temperatures and high UVB exposure can drive rapid evolution (Rhode 1992; Davies *et al*. 2004; Dowle *et al*. 2013; Gillman *et al*. 2014; but see Bromham & Cardillo 2003). Drivers of molecular evolution can impact trait evolution (Davies & Savolainen 2006), although it is not clear whether nor how rates of molecular and phenotypic change are related (Bromham *et al*. 2002). Indeed, few studies test the impacts of factors associated with increased genetic variability and fixation rates on trait macroevolutionary rates (but see Cooper & Purvis 2009).

In contrast, biotic interactions have received much attention, particularly as drivers of rate variation in ecomorphological traits (e.g. Drury *et al*. 2018). Antagonistic interactions between species can accelerate trait evolution if lineages rapidly differentiate in key traits to avoid competition (Grant & Grant 2006). Accordingly, secondary sympatry has been linked with high evolutionary rates via character displacement (Dayan & Simberloff 2005; Pfennig & Pfennig 2009; Voje *et al*. 2015; but see Tobias *et al*. 2014). The absence of competitors is also thought to drive rapid evolution, as species diverge to exploit free resources. Indeed, isolated environments, especially islands, have long been hypothesised as drivers of rapid diversification and phenotypic evolution (Losos & Ricklefs 2009).

At deep‐time scales, patterns of phenotypic accumulation have mostly been linked to the potential to explore novel ecological resources, and also to the feedbacks of species packing on morphological diversification (Hunter 1998; Mahler *et al*. 2010; Rabosky & Adams 2012; Weir & Mursleen 2013). Heterogeneity in evolutionary rates has been described as a mixture of rapid evolutionary episodes generating large morphological differences between sister‐clades, and phases of gradual, cumulative change as species diverge and adapt to the niche invaded by their common ancestor (Simpson 1953; Uyeda *et al*. 2011; Cooney *et al*. 2017; Landis & Schraiber 2017). It is debated how episodes of rapid evolution should affect subsequent evolution of descendants (recently reviewed in Rabosky 2017). Bursts of evolution that mark clade‐wide shifts towards unique morphologies are thought to associate with access to novel ecological resources and rapid evolution of descendants (Hunter 1998; Losos 2010; Losos & Mahler 2010). Alternatively, evolution of morphologically distinct lineages might inhibit subsequent divergence when there are adaptive (Wright 2017) or developmental (Felice & Goswami 2018) constraints on phenotypic change, and also if distinctiveness links to specialisation to a narrow set of resources (Collar *et al*. 2009). The number of species accumulating within clades is also linked to phenotypic evolution and to the distinctiveness of ancestral phenotypes (Ricklefs 2004), and morphological distinctiveness has been associated with species‐poor clades (Ricklefs 2005). Clade species richness impacts the rate of trait change because with more species, the potential for biotic interactions among closely related (and ecologically similar) species increases. Also, as the niche occupied by the ancestral phenotype fills with species and the potential for ecological opportunity declines, the rate of trait evolution is expected to slow down (Gavrilets & Losos 2009). Alternatively, fast trait divergence is expected to expand clade morphological and ecological space (Hulsey *et al*. 2013; Weir & Mursleen 2013), and thus enable high species richness (Schluter 2001; Rundle & Nosil 2005; Jonsson *et al*. 2012).

Here, we test multiple hypotheses for explaining variation in rates of trait evolution at both deep and more recent taxonomic levels. We focus on avian beak shape, an ecologically relevant trait for which there is already evidence of high variability in rates of evolution (Lovette *et al*. 2002; Reddy *et al*. 2012; Cooney *et al*. 2017). We use an extensive data set of 3D scans of beaks from 5551 species and multivariate models to estimate rates of trait evolution. We predict that rapid beak shape evolution should be associated with aspects of species ecology (e.g. increased strength of resource competition and ecological opportunity), and with factors generally associated with rapid molecular evolution (fast life history cycles and living in highly mutagenic environments).

## Materials and Methods

### Beak shape data

We collected beak shape data for 5551 species across 193 (out of 194) bird families, sampling at least 25% of species in each bird family (except *Caprimulgidae* and *Rhipiduridae*, where data were available for only 19% of the species in the family; the full list of species and proportion of species covered in each family can be found in Appendices S1 and S2). Our 3D scanning, landmarking and geometric morphometrics analyses follow protocols in Cooney *et al*. (2017). Briefly, we used study skins from the Natural History Museum (Tring) and from the Manchester Museum collections to measure one mature individual (preferentially male, reflecting sex biases in ornithological collections) for each species. For groups where the beak is obscured by feathers (obstructing the scanning of the beak, see below), and for species with no suitable specimens in the collections, skeletal material was used instead.

We took 3D scans of bird beaks using white and blue structured light scanning (*FlexScan3D*). For each beak, we obtained 5–25 scans and used FlexScan3D (LMI Technologies, Vancouver, Canada) software to align and combine them. We used Geomagic Studio (3DSystems) to reduce each combined scan to 500 000 faces, and to remove any flaws (holes, feather excess, reversed normals, high aspect ratio spikes). The clean meshes were processed using landmark based geometric morphometrics analysis, which analyses geometric shape variation by placing homologous key points (landmarks) on Procrustes‐aligned study surfaces (Adams *et al*. 2013). We define a total of four landmarks and 75 semi‐landmarks, which were slid to reduce bending energy (see Cooney *et al*. 2017 Extended Data Fig. 1). The four landmarks were as follows: (1) the tip of the upper beak, and the posterior margin of the upper beak on the (2) dorsal midline profile, (3) left and (4) right tomial edges. The 75 sliding semi‐landmarks constitute the dorsal profile (joining points 1 and 2), and the left and right tomial edges (curves joining point 1 to points 3 and 4 respectively). Landmarking was performed by the authors (63% of total markups) and by members of the public on the MarkMyBird crowd‐sourcing website (http://www.markmybird.org). Each beak was marked by at least three independent users (over 20 000 markups in total). We used R scripts to quality control the data. A landmarking effort was considered unsuitable if: (1) the left and right tomial edges were inversed or placed asymmetrically, (2) the semi‐landmarks along the left and right tomial edges were placed in the incorrect order or did not correctly follow the curve of the beaks and (3) there was a large discrepancy in the position of equivalent landmarks between different users (between‐users Procrustes distance ≥ 0.2). Using this crowd‐sourcing approach for landmarking avian beaks produces reliable results, as landmarks show a high repeatability between users (Cooney *et al*. 2017). We used the R package Geomorph (Adams *et al*. 2017) to process the user‐averaged beaks shape of each species via geometric morphometrics analysis. Here we focus on beak shape, as it represents a key axis of ecomorphological differentiation between major avian groups (Cooney *et al*. 2017). While size is also a major axis of ecomorphological differentiation, shape is more indicative of how a structure functions biomechanically and functionally, with size simply scaling that function. Furthermore, differences in size tend to overwhelm differences in shape, which is particularly problematic when shapes are highly disparate (as here) because dramatically different shapes may have the same centroid size (Zelditch *et al*. 2012). We therefore first removed the effects of size, translational and rotational position on landmark configurations by performing a Generalized Procrustes Analysis. We then extracted the main axes of shape variation via a PCA and phylogenetic PCA analysis (pPCA, Revell 2009). The latter is designed to account for potential biases in the PCA analysis resulting from the non‐independence in phenotypes between species caused by shared ancestry (Revell 2009; Polly *et al*. 2013; Uyeda *et al*. 2015).

**Figure 1:**
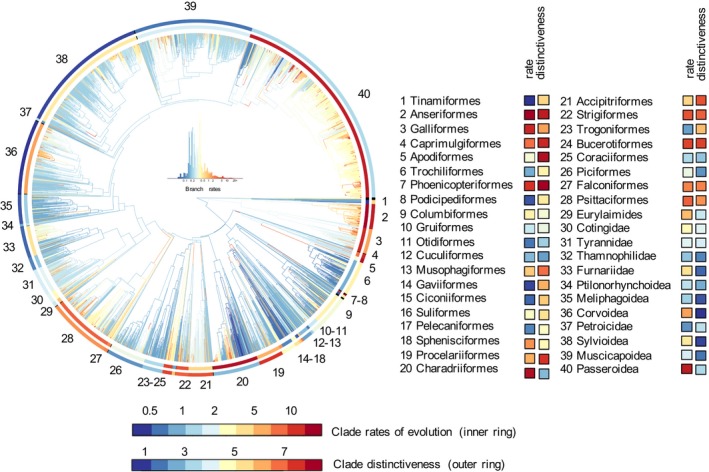
Multivariate rates of beak shape evolution. Branches are coloured by the median multivariate rate of evolution. Rings are coloured by clade rates of evolution (inner ring) and clade morphological distinctiveness, that is the Euclidean distance between the centre of the clade and the overall centre of the morphospace (outer ring). Black indicates clades smaller than five species, which were not included in the clade‐level analyses.

### Phylogenetic data

To assess the impacts of phylogenetic uncertainty we used phylogenetic tree distributions from http://www.birdtree.org (Jetz *et al*. 2012) to generate consensus trees. We sampled 10 000 Hackett backbone (Hackett *et al*. 2008) ‘stage 1’ trees (i.e. trees including only species for which genetic data are available) and ‘stage 2’ trees (i.e. trees with all 9993 species). We then pruned the sampled trees to generate distributions for species in our data set (5551 species out of which 4108 species had genetic data). We used Tree‐Annotator (Drummond *et al*. 2012) to generate maximum clade credibility trees and used two alternative methods to infer branch lengths by setting node heights (1) equal to ‘common ancestor’ node heights and (2) equal to the heights of the target tree. In addition, we used the recently published avian phylogeny from Prum *et al*. (2015) to build alternative consensus trees for our list of species. We followed Cooney *et al*. (2017) to merge the species level resolution of Jetz *et al*. (2012) to the backbone phylogeny derived from Prum *et al*. (2015) and build maximum clade credibility trees. A list of all alternative trees and data sets used to perform the multivariate rate analyses is given in Table S1.

### Rates of beak evolution

We estimated rates of beak shape evolution using the variable rates model (VarRates command) in the software BayesTraits, version 2 (available from http://www.evolution.rdg.ac.uk/), which uses a single tree and allows for the analysis of multivariate traits. The model was run setting uniform (default) priors, with no restrictions for the phylogenetic mean (alpha) and Brownian variance (sigma), and allowing correlation between variables. While in principal PCs are orthogonal, hence uncorrelated multivariate runs would be justified (Cooney *et al*. 2017), we used a more flexible approach and allowed for correlation between variables as it could account for potentially weak correlations between the PCs that emerge due to phylogenetic history. Allowing for non‐independence between variables, alongside the large ratio of species to target traits minimises potential biases in multivariate trait analyses (Adams & Collyer 2018). Each run was set for least 2 billion iterations (running for ~6 months on a 2.30 GHz Linux machine), sampling every 100 000 iterations, with a 50% post burn‐in. For each tree, we ran the models with the PC and pPC axes (i.e. traits) that explained 99% variation in beak shape. Runs were set up at least once (Table S1), and a potential scale reduction factor smaller than 1.1 was considered as an indicator of between‐chains convergence (i.e. the Gelman‐R diagnostic; R package CODA, Plummer *et al*. 2006). Within chain convergence was assessed using trace and auto‐correlation plots, alongside effective sample size (values ≥ 200 were taken as indicators of chain convergence; Plummer *et al*. 2006).

The multivariate variable rates model allows for heterogeneity in rates of evolution by scaling both single branches and a target branch plus its descendants at any location in the tree (Venditti *et al*. 2011). We summarise alternative configurations of rate scaled trees by calculating the median rate of evolution for each branch across the posterior. We used tip rates (i.e. rate values on the tip branches) as a measure of species‐specific rates of beak shape evolution. We calculate rates of evolution using the number of PC and pPC axes that explained 99% of beak shape variation.

To predict patterns of phenotypic accumulation across deep‐time scales, we also calculated rates of evolution for well‐defined, monophyletic groups of species (Jetz *et al*. 2012). We split the non‐passerines into orders, and passerines into well‐supported families and superfamilies (Jetz *et al*. 2012). We pruned the consensus trees to include only species belonging to clades with at least five representatives and used transformPhylo.ML in the R package MOTMOT (Thomas & Freckleton 2012) to calculate multivariate relative clade rates of evolution. We report results from fitting multivariate models of trait evolution on the PC scores in the main text, and the results from the rate‐analysis using pPC scores in the supplement (Table S5, Table S6).

### Correlates for rates of beak shape evolution

We used species body mass (g) from Elton Traits (Wilman *et al*. 2014). We used the average age of parents as an estimate of the turnover of generations, or generation length (BirdLife International (2018) IUCN Red List for birds. Downloaded from http://www.birdlife.org on 05/05/2017). We used species' distribution maps from BirdLife International (BirdLife International and Handbook of the Birds of the World (2016) bird species distribution maps of the world. Version 6.0. Available at http://datazone.birdlife.org/species/requestdis), considering only native species and also subsetting ranges to breeding areas where species are highly probable or known to occur. We used these maps to calculate: (1) species' range sizes, (2) the proportion of species' ranges that occurs on islands, (3) mean annual temperature (Hijmans *et al*. 2005), (4) mean annual UVB levels (Beckmann *et al*. 2014), and (5) an index of potential competition. The index of potential competitors was calculated by dividing species' distribution ranges into equal area grid cells (resolution of ~110 km), and counting the number of confamilial species that share diet and foraging strategy with the focal species in each grid cell (based on EltonTraits, Wilman *et al*. 2014). We then averaged these values across grid cells. The spatial data handling was done using the R packages letsR (Vilela *et al*. 2015) and raster (Hijmans & Etten 2012). We controlled for species' age by including the length of the branch leading to each species in the consensus tree. Lastly, we included the mean Procrustes distances between users marking each beak to account for user error (referred to as measurement error).

### Species level analyses

We used PGLS analysis (Grafen 1989; Martins & Hansen 1997) in the R package caper (Orme *et al*. 2013) to correlate species‐specific rates of evolution with the potential drivers for rate variation described above. We also ran the analyses with species' clade included as an interaction term, to estimate whether and how the relationship between rates and potential explanatory variables changes in specific clades. Furthermore, migratory birds will likely spend most of the annual cycle in their non‐breeding ranges. To account for potential biases of using breeding ranges only, we also performed the analyses including migratory status as an interaction term (i.e. full migrant vs. resident as described in BirdLife International (2018) IUCN Red List for birds. Downloaded from http://www.birdlife.org on 05/05/2017).

### Clade‐level analyses

We measured the distinctiveness of each clade in beak shape morphospace by calculating the Euclidean distance between the centre of the clade and the overall centre of the morphospace using the PCs that explained up to 99% of beak shape variation (Fig. S1). Longer distances imply more peripheral clades with greater potential ecological opportunity. We correlate this measure with clade rates of evolution, also considering species richness (the total number of species in each clade), an index of the potential strength of competition in the clade (by averaging the species‐specific competition index estimated above), clade age (age of its most recent common ancestor) and controlling for the proportion of island species in each clade, and the average range size for species in the clade. We logged body mass, generation length, range size, number of competitors, beak distinctiveness and age to ensure normal distribution of predictors. We used variance inflation factors to test the independency of predictors.

## Results

### Patterns of beak shape evolutionary rates

The first eight PC axes from the PCA analysis explained 99% of variation in beak shape, with almost half of this variation being explained by PC1 (variation from a long, narrow beak to a short, wide beak; Fig. S2, Table S2). Some species and clades of species show extreme PC values on one or two axes (e.g. *Anseriformes*,* Bucerotiformes*), whereas others consistently show extreme PC scores on multiple axes, marking major deviations from the general cone‐like beak shape (e.g. *Phoenicopteriformes*,* Apodiformes*; Fig. S2).

We find evidence of extensive variation in evolutionary rates, both among tip and internal branches (Fig. 1). Tip rates in particular show a high degree of skewness, and examples of exceptionally high species‐specific rates are mostly associated with the evolution of very unusual beaks, for example we see extreme PC and rate values for the laterally curved beaks of *Anarhynchus frontalis* (wrybill) and the *Loxia* genus (crossbills). We find several internal single‐lineage high rates of evolution including several major shifts that were not detected by Cooney *et al*. (2017). These mark the evolution of lineages towards the periphery of the beak shape morphospace (e.g. *Strigiformes*,* Bucerotiformes*,* Accipitriformes*,* Phoenicopteriformes*,* Psittaciformes*), and can coincide with major differences in morphology between descendant sister‐clades. Similar to species‐specific evolutionary rates, we find great variation in evolutionary rates between broadly recognised clades of species (Fig. 1). We see high rates of evolution in some large passerine groups with generally average beak types (e.g. *Passeroidea*,* Sylvioidea, Corvoidea*). Many non‐passerines groups also have high rates of evolution, but most of these are clades with unique beak shapes, several of which are also species poor (e.g. *Phoenicopteriformes*; Fig. S8b).

### Correlates of species‐specific rates of evolution

**Figure 2:**
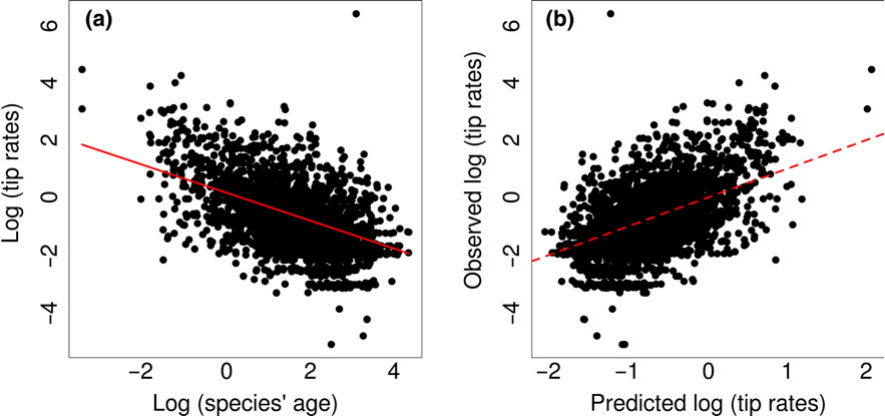
(a) The relationship between species‐specific rates of evolution and species' age, *P* < 0.001, *R* ^2^ = 0.20. (b) The relationship between the observed and predicted rate of evolution by the full PGLS model: adj. *R* ^2^ = 0.21. The dashed line indicates the 1 : 1 line of predicted vs. observed values.

We find a strong negative effect of species' age on species‐specific rates of evolution, with age alone explaining 20% of variation in tip evolutionary rates (Fig. 2a). In addition to age, the full model identifies significant positive effects of proportion of range occurring on islands (Fig. S3a), UVB levels (Fig. S3b), and measurement error (Fig. S3c). However, the effect sizes and variation explained by these variables are small (Table 1). We find no effect of life history traits, range size or number of competitors on tip rates of evolution (Table 1, Fig. S4), and overall almost 80% of variation in species‐specific evolutionary rates remained unexplained (Fig. 2b). In resident species, both temperature and UVB levels have a weak, negative effect on evolutionary rates (Fig. S6). There is no significant effect of climatic variables on rates of evolution for migratory species. When including clade as an interaction term in the model, we largely recover the same trends observed in the main model (Table S4, Fig. S5).

**Table 1:** Correlates of species‐specific rates of evolution; λ = 0.626, d.f. = 9,3734, adjusted *R* ^2^ = 0.21. Stars indicate levels of significance: P < 0.001 (***), 0.001 < P < 0.01 (**), and 0.01 < P < 0.05 (*)

Predictor	Slope ± SE	*t*	*P*
Log species' age	−0.478 ± 0.010	−30.186	< 0.001***
Log body mass	0.040 ± 0.022	1.794	0.073
Log generation length	0.103 ± 0.080	1.294	0.196
Mean annual temperature	−0.002 ± 0.002	−0.669	0.504
Mean annual UVB levels	0.000 ± 0.000	−2.195	0.028*
Log range size	−0.009 ± 0.006	−1.379	0.168
Proportion of island range	0.115 ± 0.046	2.488	0.013*
Log number of competitors	0.004 ± 0.013	0.271	0.786
Measurement error	0.917 ± 0.420	2.184	0.029*

### Correlates of clade rates of evolution

**Figure 3:**
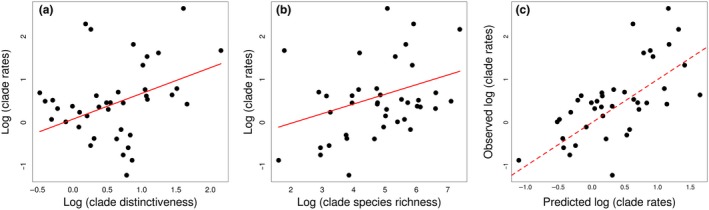
The relationship between clade rates of evolution and (a) clade beak distinctiveness, *P* < 0.001, (b) clade species richness, *P* < 0.001. (c) The relationship between the observed and predicted clade rates of evolution by the full PGLS model: adj. *R* ^2^ = 0.52. The dashed line indicates the 1 : 1 line of predicted vs. observed values.

Clade evolutionary rates were positively correlated with clade beak distinctiveness and species richness (Fig. 3a,b, Table 2). We also find a weak negative effect of potential competition strength on evolutionary rates (Fig. S7a). The number of species in clades relates negatively with the distinctiveness of their phenotype, and clades with distinct beaks are typically species poor (Fig. S8). Together, these factors explain just above half of the variation in clade rates of evolution (Fig. 3c). Clade age does not have a significant impact on rates. However, using a finer split of species in clades with more variability in clade age, we recover a negative impact of clade age, alongside the effects of beak distinctiveness and richness on clade rates (Fig. S9, Fig. S10, Table S7). The results we find at species and clade levels are generally robust to alternative avian phylogenies, methods for building a consensus tree, and the inclusion of species without genetic data (Table S3, Table S5, Table S6, Fig. S11).

**Table 2:** Correlates for clade rates of evolution; λ = 1.000, d.f. = 6,33, adjusted *R* ^2^ = 0.52. Stars indicate levels of significance: P < 0.001 (***), 0.001 < P < 0.01 (**), and 0.01 < P < 0.05 (*)

Predictor	Slope ± SE	*t*	*P*
Log clade age	−0.077 ± 0.348	−0.221	0.827
Log clade beak distinctiveness	0.881 ± 0.227	3.882	< 0.001***
Log clade species richness	0.501 ± 0.119	4.218	< 0.001***
Log average range size	−0.157 ± 0.081	−1.933	0.062
Proportion of island species	−0.322 ± 0.841	−0.383	0.704
Log average number of competitors	−0.376 ± 0.179	−2.097	0.044*

## Discussion

Here, we describe heterogeneity in rates of avian beak shape evolution and find considerable variation in the rate of phenotypic change among both species and clades (Fig. 1). We see several instances of rapid major morphological differentiation between sister‐clades, consistent with Cooney *et al*. (2017). When such events place clades at the periphery of the eco‐morphospace, we see subsequent high rates of evolution of descendants. Rapidly evolving groups are not necessarily distinct, however, and several clades with average (i.e. non‐peripheral) beak types also show high rates of evolution.

We find that variation in species‐specific rates of ecomorphological traits evolution is difficult to predict, and after controlling for species age, the factors we considered associate weakly with evolutionary rates. Species' age correlates negatively with rates of phenotypic change, as expected under speciational trait evolution followed by stasis. We note, however, that phylogenetic and/or measurement error can also cause an over‐inflation of trait evolutionary rates that is particularly prevalent for young species (Rabosky 2015). We found little to no effect of life history, environmental mutagenic factors or range sizes on species‐specific trait evolutionary rates (Table 1). UVB levels (and temperature values for resident species only, Fig. S6) weakly associate with evolutionary rates (Fig. S3b). However, the trend we note is negative, that is opposite to predictions based on the mutagenic effect of high temperatures and UVB levels (Rhode 1992). If anything, such a relationship might reflect the effect of environmental instability on rates. That is colder and UVB‐poor environments (e.g. at higher altitude or latitude) may be associated with high prevalence of fragmented and unstable range sizes, further thought to inflate evolutionary rates (Liu *et al*. 2006; Martin *et al*. 2010; Flenley 2011; Lawson & Weir 2014). Thus overall, our candidate factors associated with an increase in the potential for genetic variability or speed of mutation fixation show little to no impact on ecomorphological rates of evolution (but see Cooper & Purvis 2009).

We also tested whether rates of evolution link to potential for competition. We find that most species do not overlap geographically with close relatives that also share foraging strategies and diet, and accordingly, we find no link between number of potential competitors and evolutionary rates (Fig. S4). Furthermore, we recover high evolutionary rates in many famous insular radiations in beak shapes, including Galapagos finches, Hawaiian honeycreepers, birds of paradise, flowerpeckers and also select parrots, white‐eyes and starlings (Fig. 1). In general, however, island species exhibit both slow and fast evolutionary rates, and within the same clade, mainland species can have similar rates to those on islands. Consequently, we find that the effect of islands on evolutionary rates is limited to several small‐scale exceptional radiations, and has a relatively small impact on the accumulation of phenotypic diversity across a global radiation such as Aves. Overall, our results imply that species‐specific ecomorphological rates of evolution are likely contingent on chance events, and hence difficult to predict across global radiations.

Rates of evolution were more predictable at the clade level. We found that the distinctiveness of clade phenotype and its species richness act additively to explain half of the variation in clade evolutionary rates. Specifically, we find that clades that occupy the periphery of the morphospace have high rates of evolution (Fig. 3a), in agreement with the idea that adaptation to a novel set of ecological resources can drive rapid phenotypic differentiation (Price *et al*. 2010; Martin & Wainwright 2011; but see Wright 2017). The effect of evolution towards the periphery of the morphospace is analogous to Simpsonian jumps to new adaptive zones, also hypothesised to drive subsequent rapid evolution via increased ecological opportunity (Simpson 1953). We also see a negative relationship between clade species richness and the distinctiveness of their beak shapes (Fig. S8b), supporting the hypothesis that lineages that evolved to exploit specialised (and thus potentially limited) resources are not expected to proliferate (Ricklefs 2005). These results imply that ecological opportunity in the form of evolution towards unique phenotypes is an important driver of rapid evolution, but the peculiarity of the ancestral phenotype constrains the prospective number of, and disparity among, descendants.

Species richness and trait evolutionary rates are, however, also positively linked (Fig. 3b), and passerines in particular represent fast evolving, species‐rich clades with generally average beak types. There is mixed evidence that trait evolutionary rates correlate with diversification and species richness (Adams *et al*. 2009; Burbrink *et al*. 2012; Rabosky & Adams 2012; Rabosky *et al*. 2013; Igea *et al*. 2017), and moreover the causality of these relationships remains unclear. Species‐rich clades are prone to intense competition for shared resources if clade members are sympatric, and could thus show fast phenotypic evolution via character displacement (Grant & Grant 2006; Davies *et al*. 2007; Martin *et al*. 2010; Freeman 2015). However, in our analyses, the vast majority of bird species show little range overlap with potential competitors (Fig. S4), and species with similar ecologies seem to be geographically isolated (consistent with a species‐sorting mechanism; Lovette & Hochachka 2006; Pigot & Tobias 2013). In clades where we do find a high proportion of species sharing ranges with ecologically similar relatives (e.g. *Trochiliformes, Tyrannidae, Thamnophilidae, Sylvioidea*), we find slower evolutionary rates (Fig. S7a); this might reflect limitations to phenotypic evolution with more species sharing the same niche (Simpson 1953). However, we interpret these results with caution, as when using a finer division of clades, the relationship is not statistically significant (Table 1, Table S7). Moreover, the negative effect between competitor numbers and evolutionary rates might be driven by multicollinearity with species richness (Table S8). We also note that our analyses focus on beak shape, but beak size (and associated allometry) is also an important axis of ecomorphological differentiation in birds, particularly within clades (Grant & Grant 2006; Bright *et al*. 2016). Removing size from the analyses thus likely reduced our power to detect an effect of biotic interactions on evolutionary rates, as in some clades competition would have been resolved by differentiation in beak size rather than shape. Overall, while species interactions can be a powerful driver of fast differentiation in (small) select radiations, our results suggest that they are unlikely to have a pervasive influence on the accumulation of beak shape variation across the global bird radiation. Recently developed methods that incorporate the effect of species interactions when modelling trait evolution will likely reveal more subtle effects of competition (Clarke *et al*. 2015; Drury *et al*. 2016), and give further insight into if and how biotic interactions link species richness with phenotypic evolution. In addition, high rates of phenotypic evolution can expand the ecological space available for species, and thus rapidly evolving clades are expected to proliferate (Schluter 2001; Rundle & Nosil 2005; Jonsson *et al*. 2012; but see Dornburg *et al*. 2011; Claramunt *et al*. 2012). Our results cannot differentiate the causality and underlying mechanism for the relationship between species richness and trait evolutionary rates.

Similar to our analyses at the species level, we account for age in our clade‐level analyses. We consider very broad taxonomic groups with little variation in age, and unsurprisingly, we find no correlation between clade age and rates of evolution. However, age correlates negatively with evolutionary rates when using a finer division of species into clades (Table S7). These results could indicate a deceleration of evolutionary rates with the packing of species in time (Agrawal *et al*. 2009; Harmon *et al*. 2010; Mahler *et al*. 2010; Lloyd *et al*. 2012; Hughes *et al*. 2013), but could also reflect the effect of measurement and/or phylogenetic error to inflate evolutionary rates for younger clades. Similar to species‐specific rates of evolution, we do not differentiate between these alternative hypotheses.

In this study, we take a comprehensive approach to explain the accumulation of ecological diversity in a major global radiation. We find little to no evidence that heterogeneity in recent evolutionary rates links to life history, environmental mutagenic factors or the presence or absence of competitors. In fact, almost 80% of variation in evolutionary rates between species remains unexplained. However, half of the variation in clade evolutionary rates is predicted by the interplay between adaptation to novel ecological resources and the number of species packing within clades. Overall, our results show that increased ecological opportunity in distinct adaptive zones is an important driver of rapid evolution, although it constraints the number of species able to pack into clades. Furthermore, we find support for the hypothesised link between rates of trait evolution and species richness, implying that rapid trait diversification is also linked with high levels of niche differentiation between related species.

## Authorship

AC, CC and GT developed the conceptual framework, devised the analytical protocols, and wrote the manuscript. AC performed the analyses. All the authors were involved in data collection and processing, and provided valuable input in writing the manuscript.

## Data Accessibility Statement

The data supporting the results (species' landmark configurations, (p)PC scores, rates of evolution, predictor variables and alternative trees) have been submitted to a Dryad repository (provisional DOI: https://doi.org/10.5061/dryad.4006fm8). The raw data (3D scans of beaks) can be accessed via NHM data portal (http://data.nhm.ac.uk/dataset/markmybird).
