# Supplementary material for: Correlates of rate heterogeneity in avian ecomorphological traits
Source: Ecol Lett. 2018 Aug 21;21(10):1505–14. doi: 10.1111/ele.13131 (PMC6175488; doi:10.1111/ele.13131)
Supplement: Supplementary file 1 [file ELE-21-1505-s001.pdf]

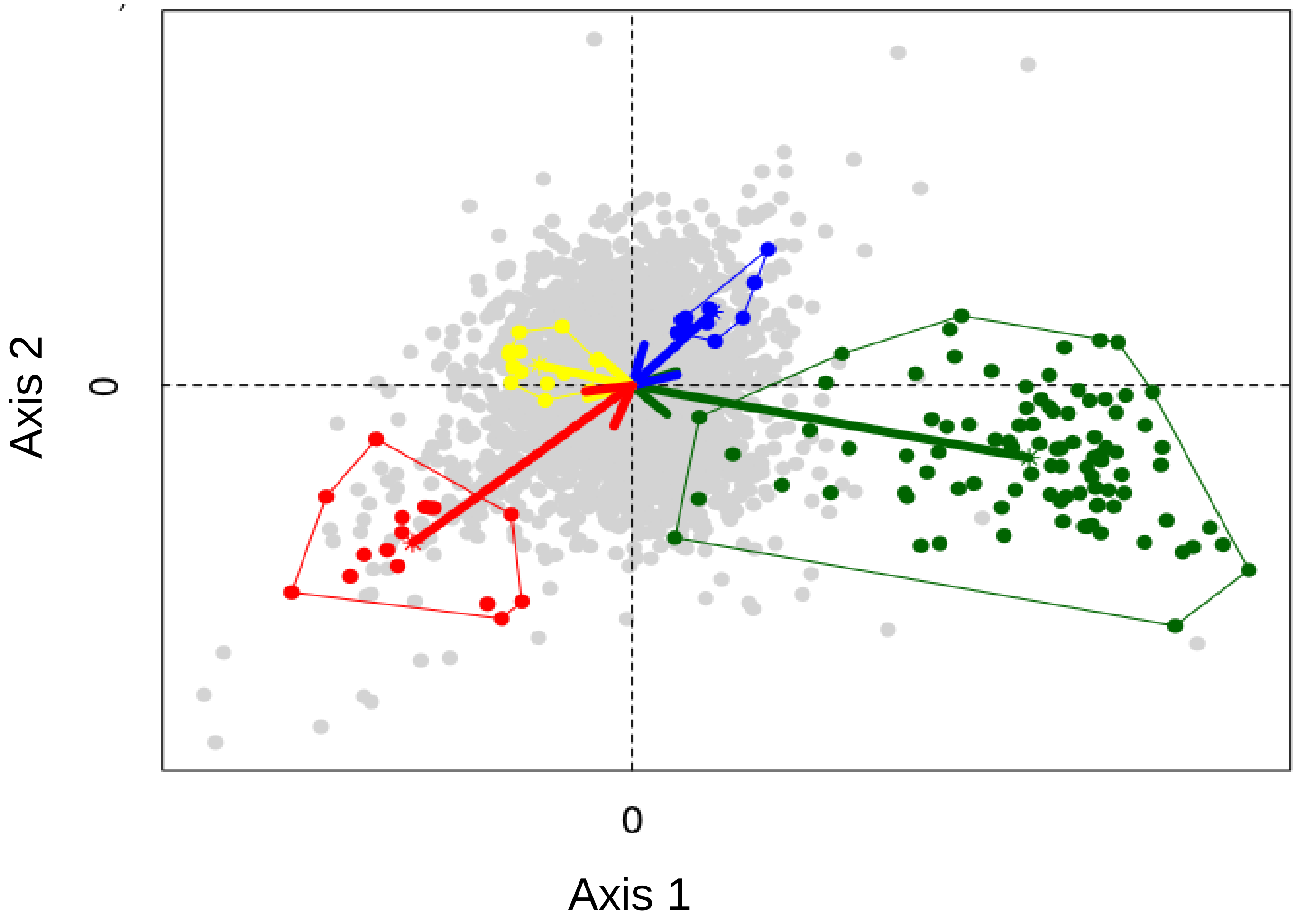

Figure S1. Illustration of how clade beak distinctiveness is calculated for target clades (delimitated here by coloured convex hulls) i.e. the Euclidean distance between the centre of the clade and the overall centre of the morphospace. The absolute value of centred PC values is considered; in our analyses we consider the first eight axes of variation when measuring beak clade distinctiveness (i.e. we calculate Euclidean distances in an eight-dimensional morphospace).

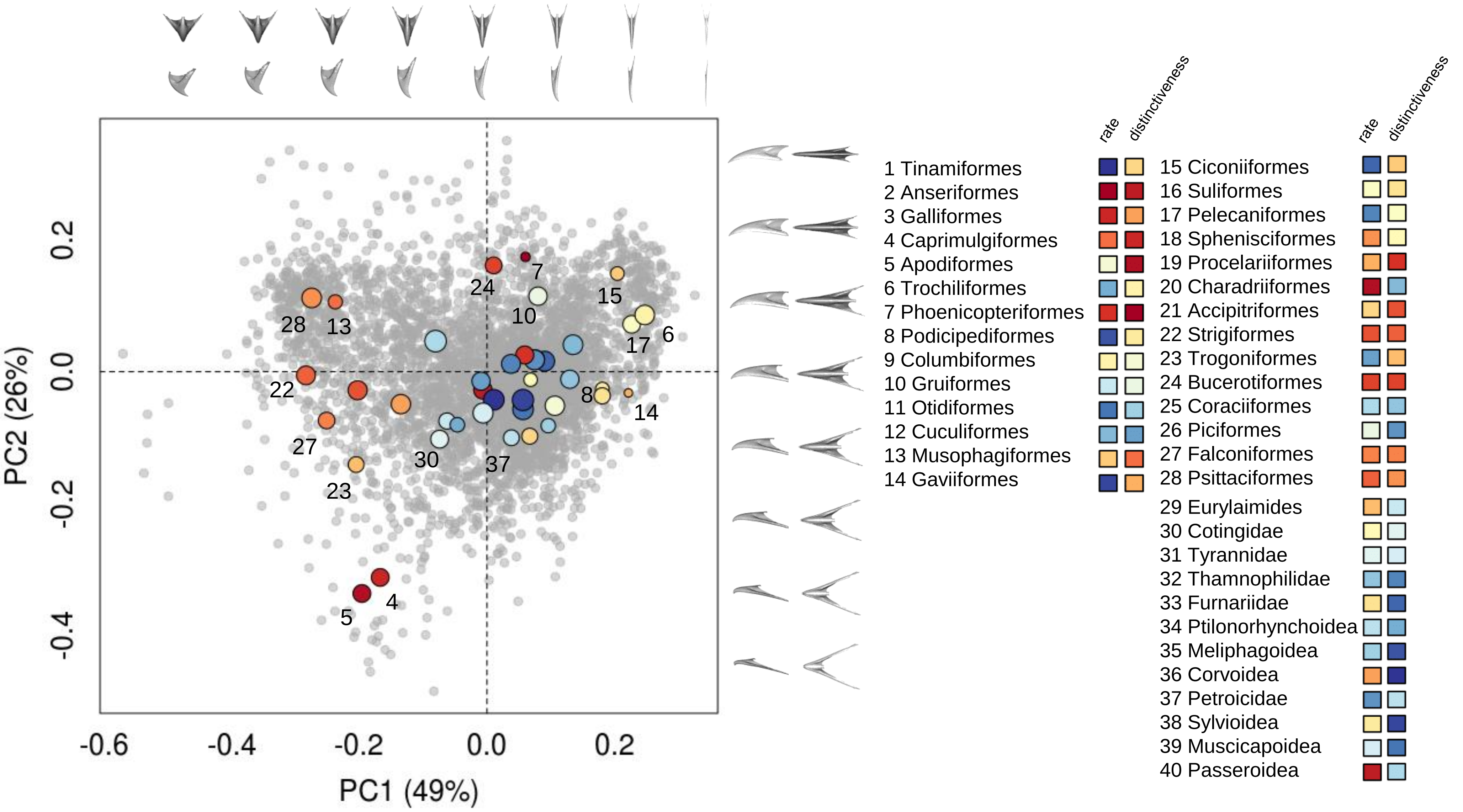

Figure S2a. Avian beak morphospace shown as a pairwise scatter plot of the 1st and 2nd PC axes (the proportion of variance explained by each PC is indicated in brackets). Warps represent the change in beak shape along each axis (top and side views). The centroid of each clade is shown, and the most distinct 10 clades on PC1 and PC2 are numbered on the plot. Centroid points are sized by the species richness of clades, and coloured by clade distinctiveness values across the first eight PC axes. Clade distinctiveness and rate of evolution are also shown in the legend.

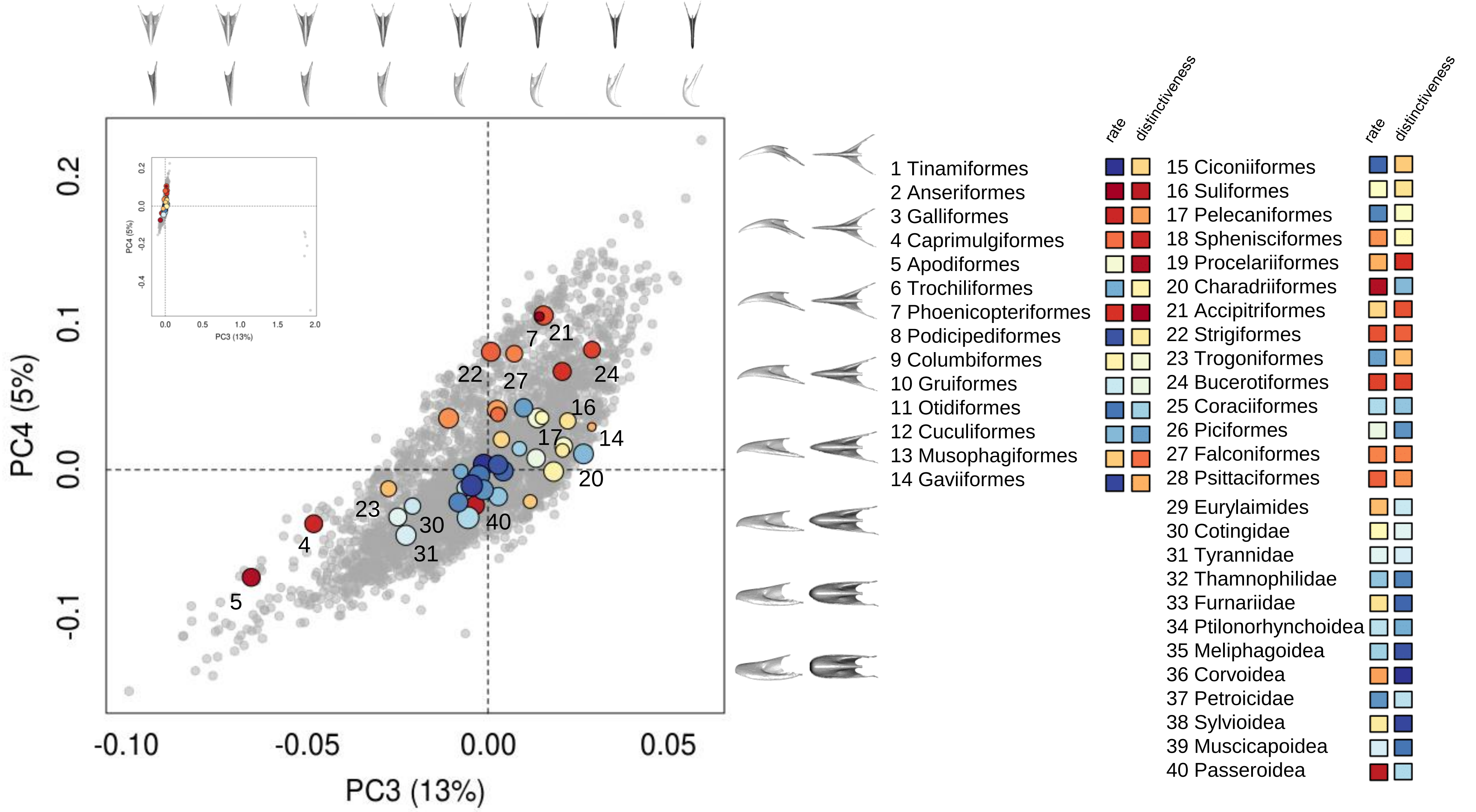

Figure S2b. Avian beak morphospace shown as a pairwise scatter plot of the 3rd and 4th PC axes (the proportion of variance explained by each PC is indicated in brackets). Warps represent the change in beak shape along each axis (top and side views). The centroid of each clade is shown, and the most distinct 10 clades on PC3 and PC4 are numbered on the plot. Centroid points are sized by the species richness of clades, and coloured by clade distinctiveness values across the first eight PC axes. Clade distinctiveness and rate of evolution are also shown in the legend. For a clearer visualisation, outstanding outlier species (i.e. PC3 > 1.5, five *Loxia* species and *Anarhynchus frontalis*) were removed from the plot; full plot given in the top-left corner.

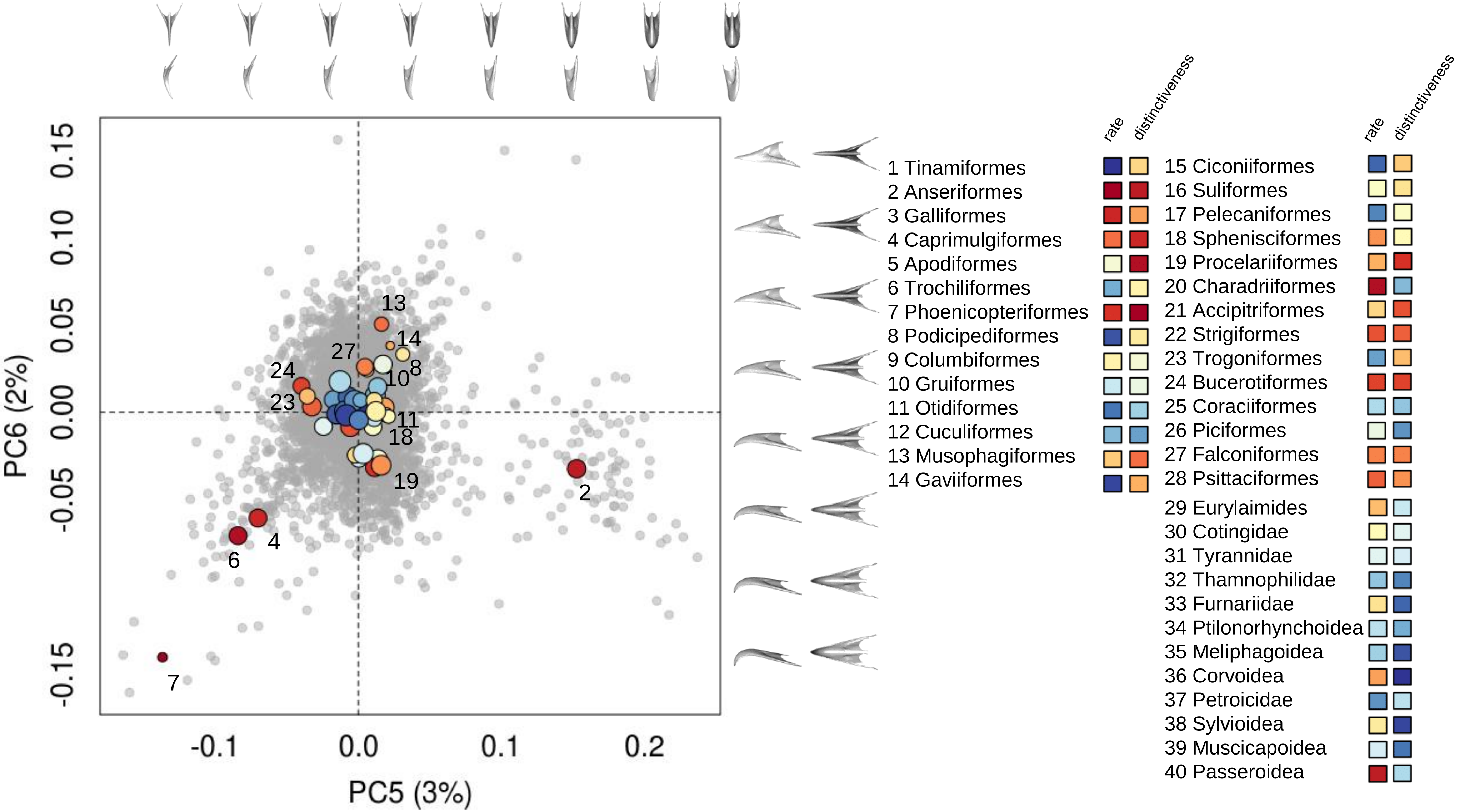

Figure S2c. Avian beak morphospace shown as a pairwise scatter plot of the 1st and 2nd PC axes (the proportion of variance explained by each PC is indicated in brackets). Warps represent the change in beak shape along each axis (top and side views). The centroid of each clade is shown, and the most distinct 10 clades on PC5 and PC6 are numbered on the plot. Centroid points are sized by the species richness of clades, and coloured by clade distinctiveness values across the first eight PC axes. Clade distinctiveness and rate of evolution are also shown in the legend.

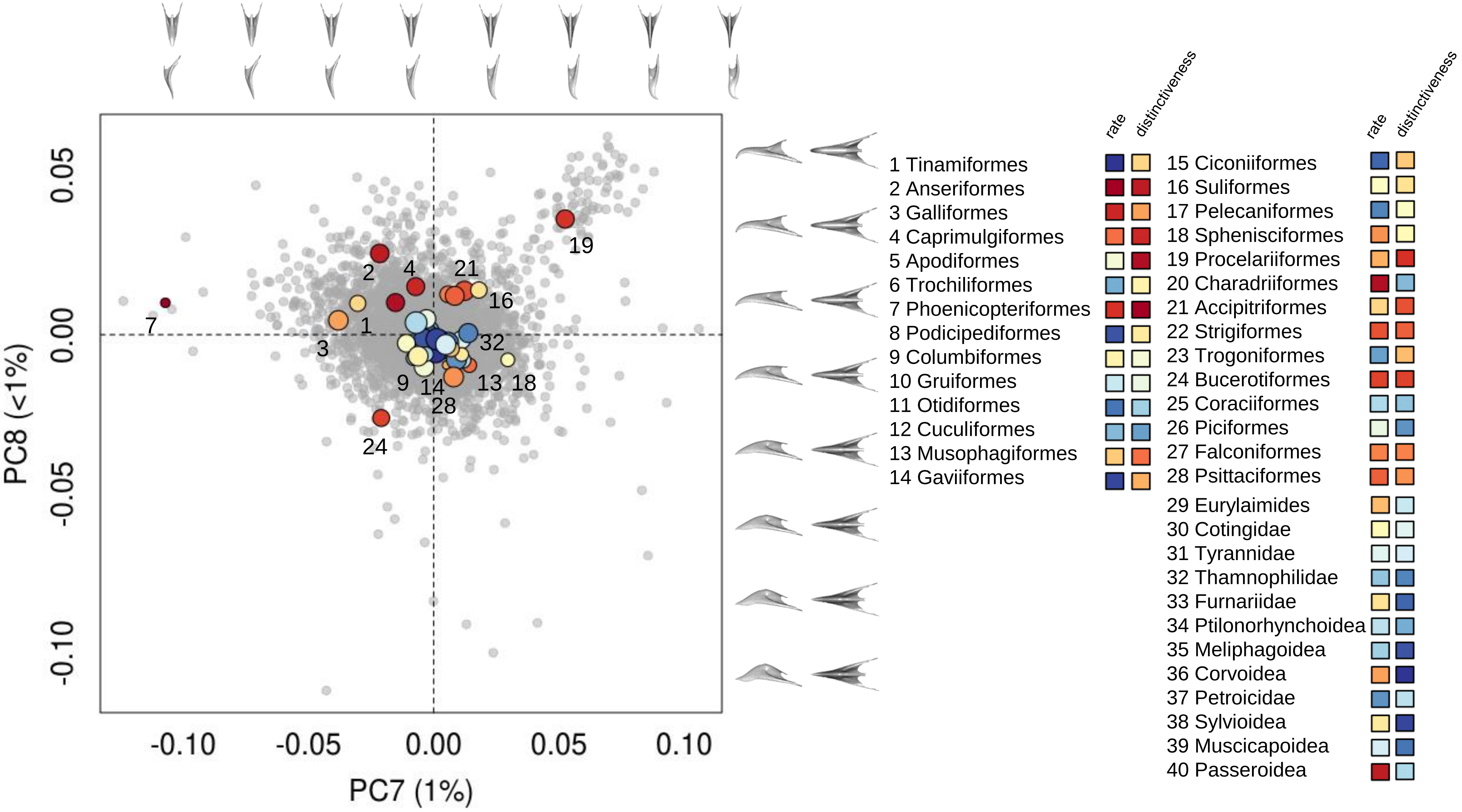

Figure S2d. Avian beak morphospace shown as a pairwise scatter plot of the 1st and 2nd PC axes (the proportion of variance explained by each PC is indicated in brackets). Warps represent the change in beak shape along each axis (top and side views). The centroid of each clade is shown, and the most distinct 10 clades on PC7 and PC8 are numbered on the plot. Centroid points are sized by the species richness of clades, and coloured by clade distinctiveness values across the first eight PC axes. Clade distinctiveness and rate of evolution are also shown in the legend.

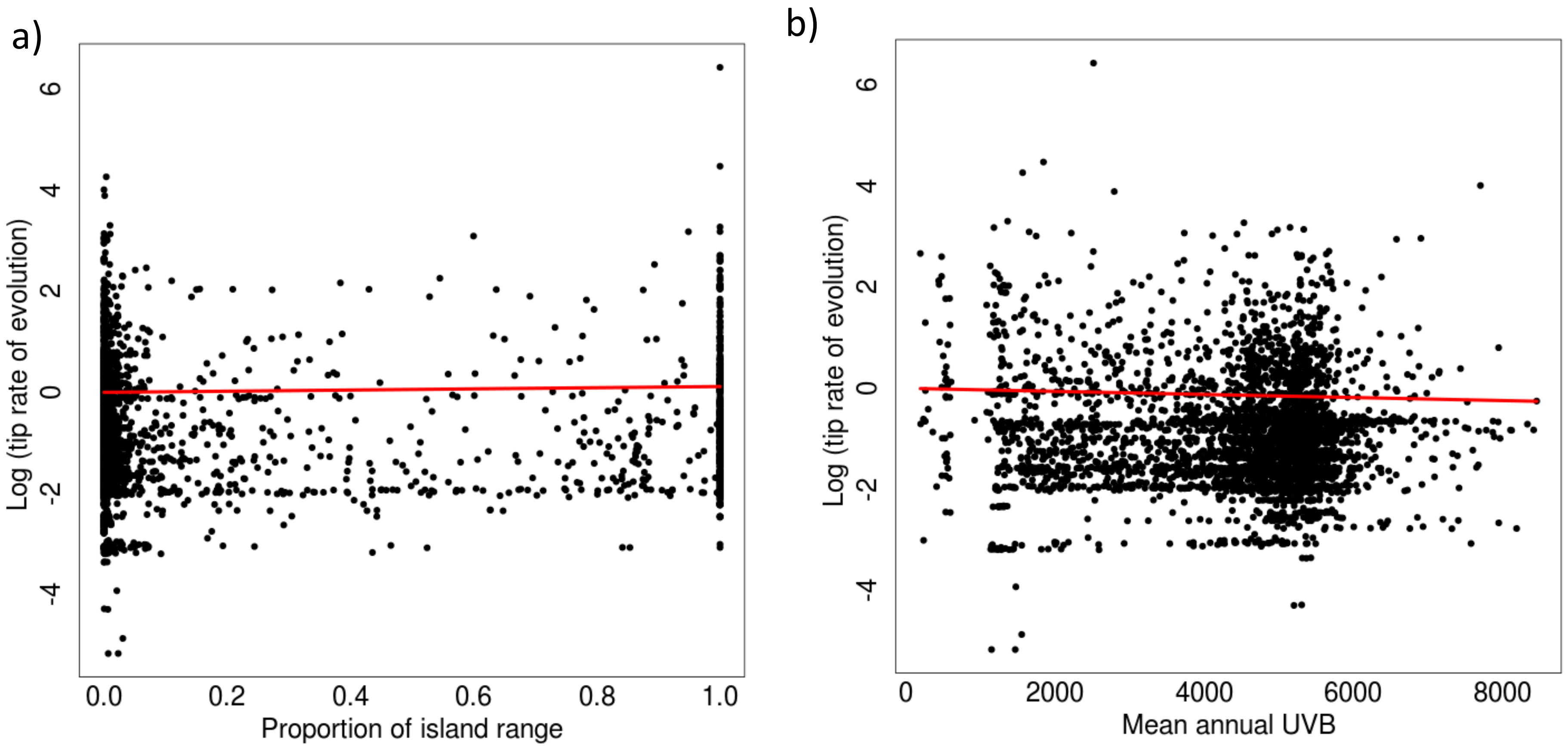

Figure S3. The relationship between species-specific rates of evolution and (a) the proportion of island range, $p = 0.013$, (b) UVB levels, $p = 0.028$.

c)

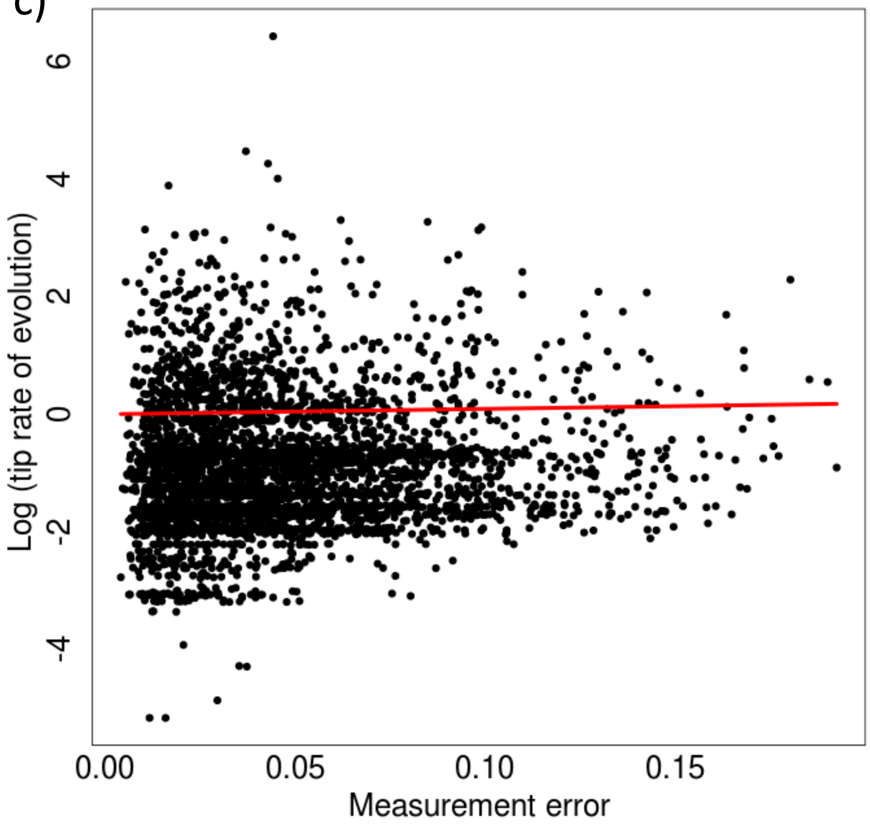

Figure S3. (c) The relationship between species-specific rates of evolution and measurement error (i.e. mean Procrustes distances between users marking each bill), $p = 0.029$.

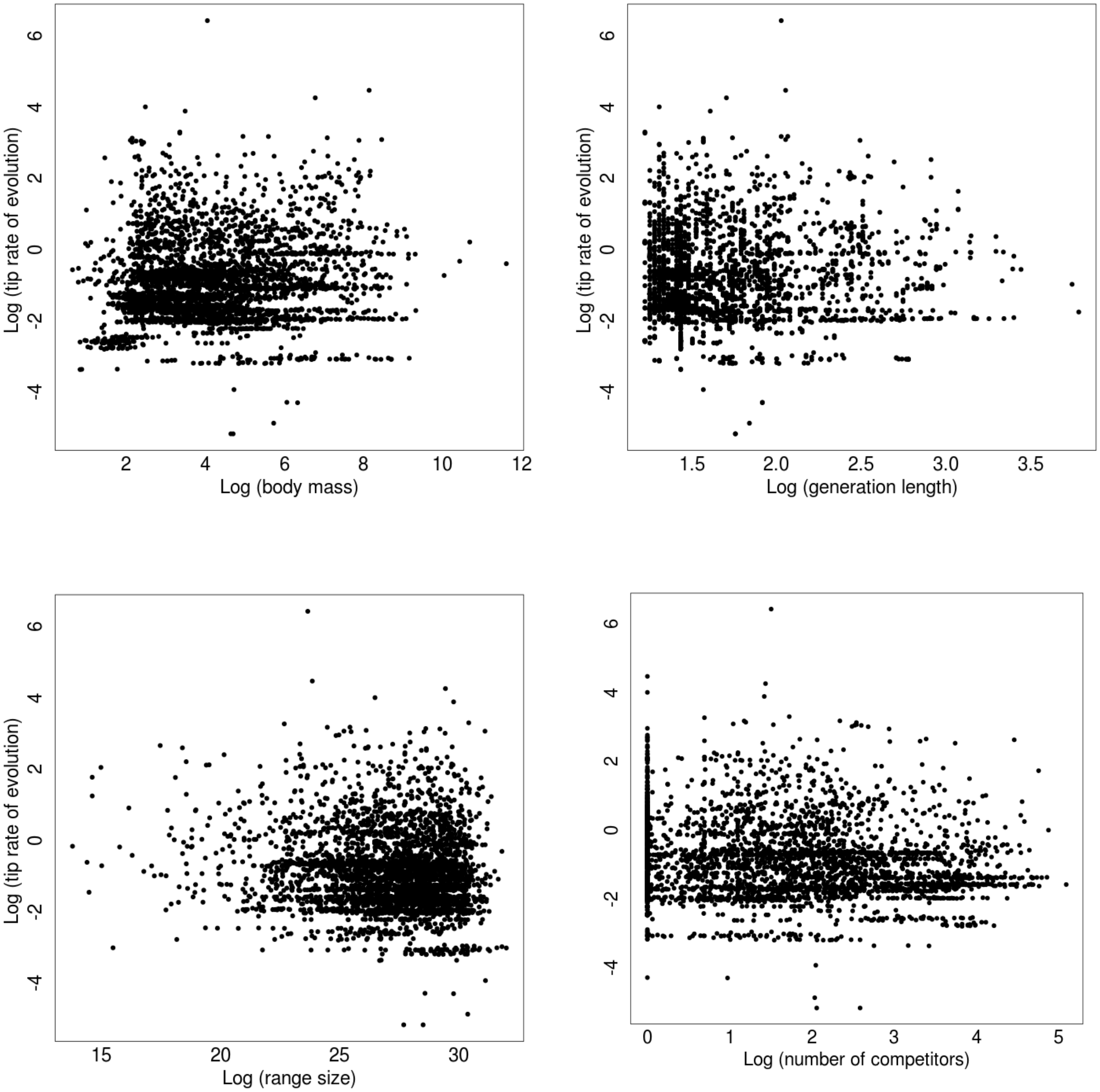

Figure S4. Correlates for species-specific rates of evolution; none of the variables correlates significantly with evolutionary rates.

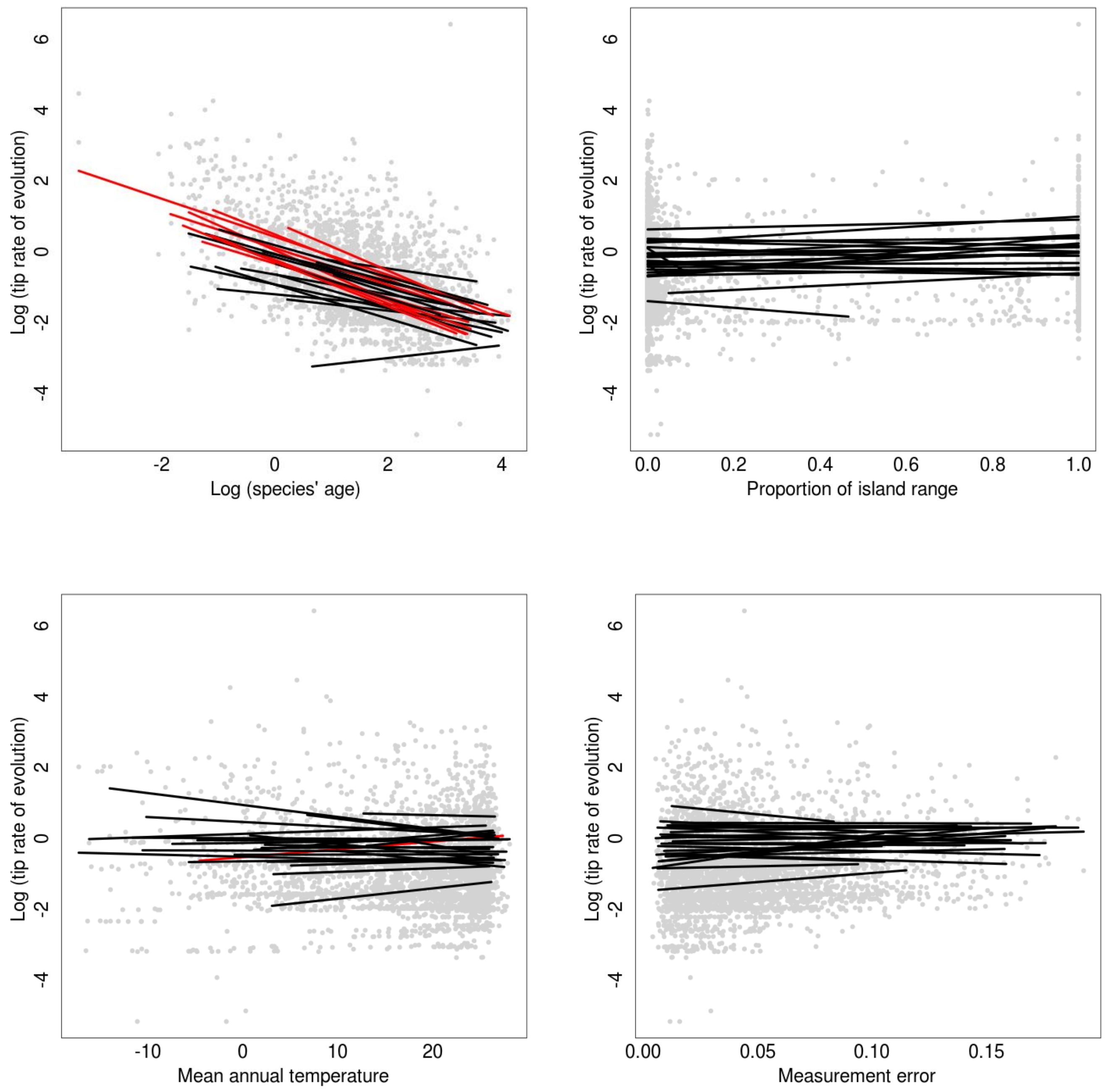

Figure S5. Correlates for species-specific rates of evolution. Each line represents a monophyletic clade of species. Red lines mark slopes for which the confidence interval does not pass 0. Continued overleaf.

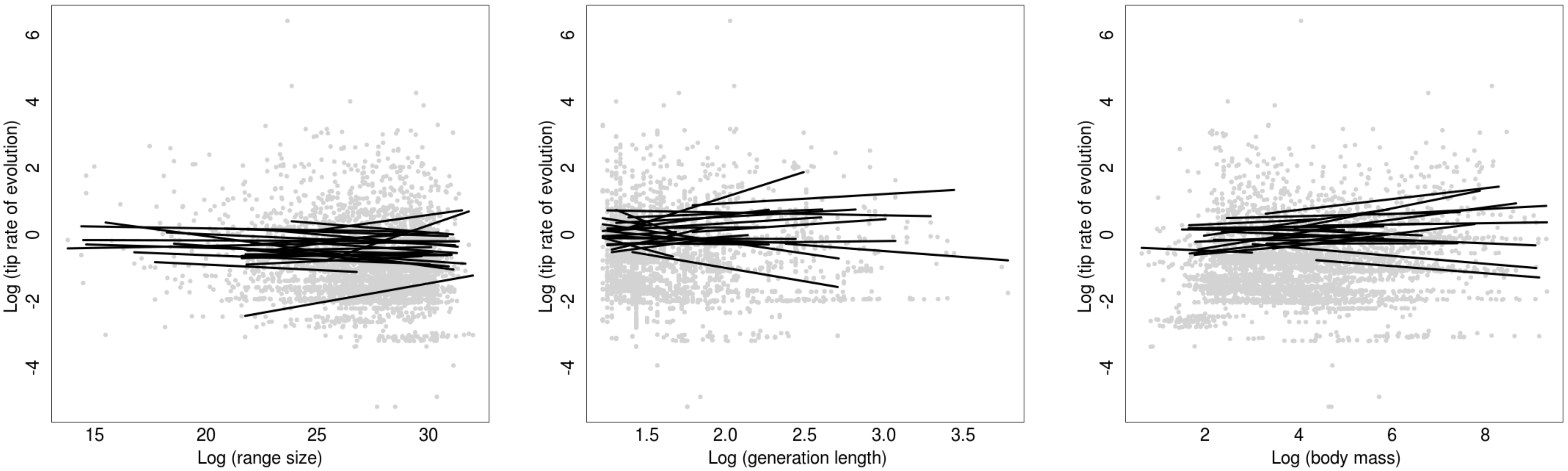

Figure S5. Correlates for species-specific rates of evolution. Each line represents a monophyletic clade of species. Red lines mark slopes for which the confidence interval does not pass 0.

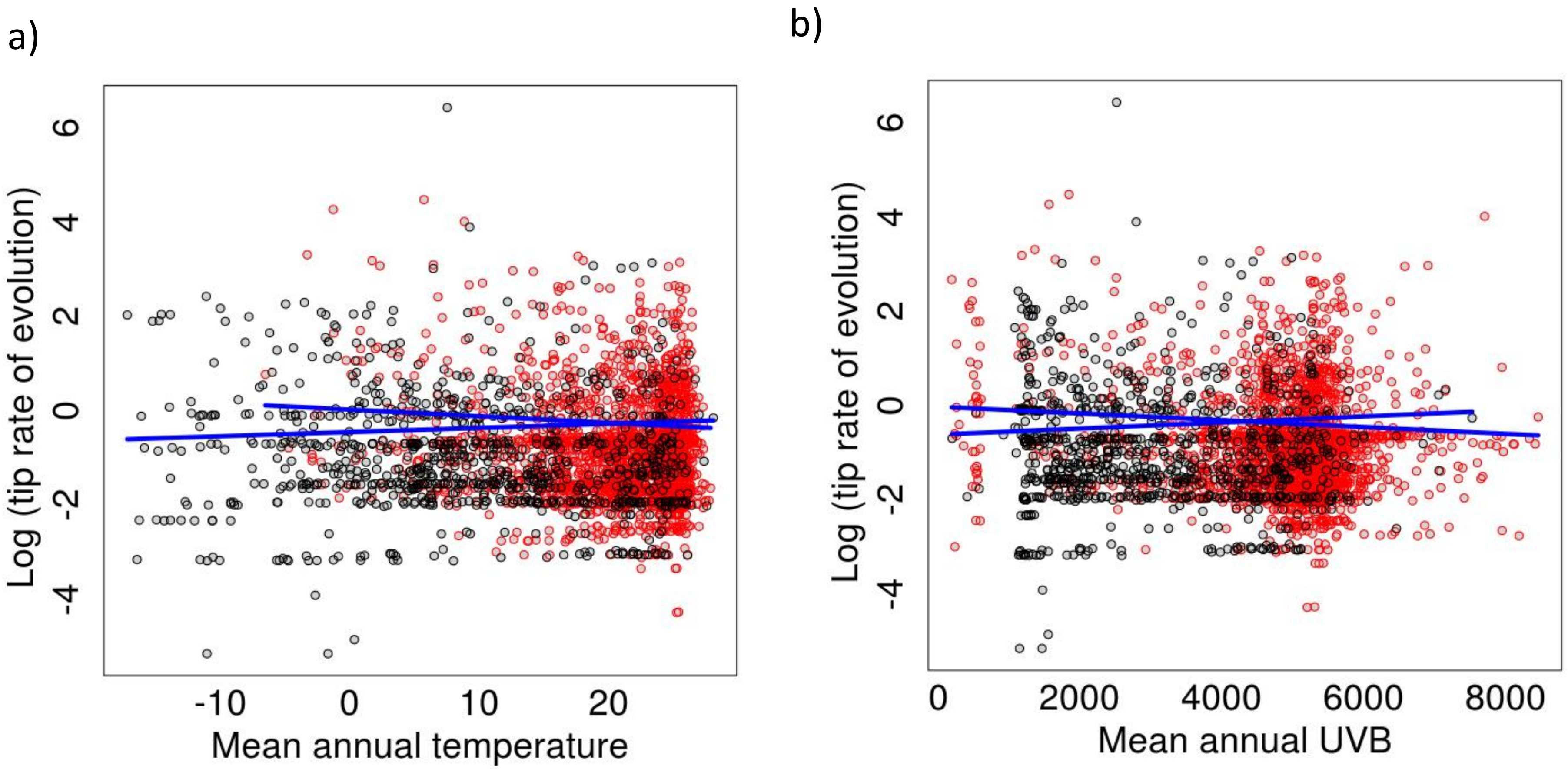

Figure S6. The relationship between species-specific evolution and (a) mean annual temperature, (b) mean annual UVB levels. Points are coloured by species' migratory status: residents (red, negative trend) vs full migrants (black).

a)

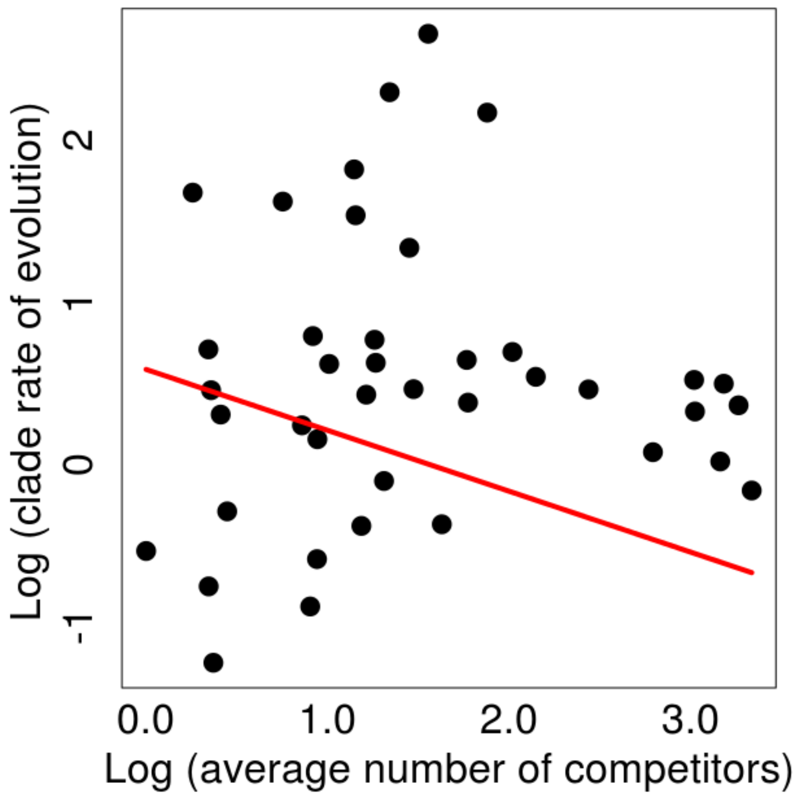

Figure S7. (a) The relationship between clade rates of evolution and average number of competitors for species in each clade, $p = 0.044$.

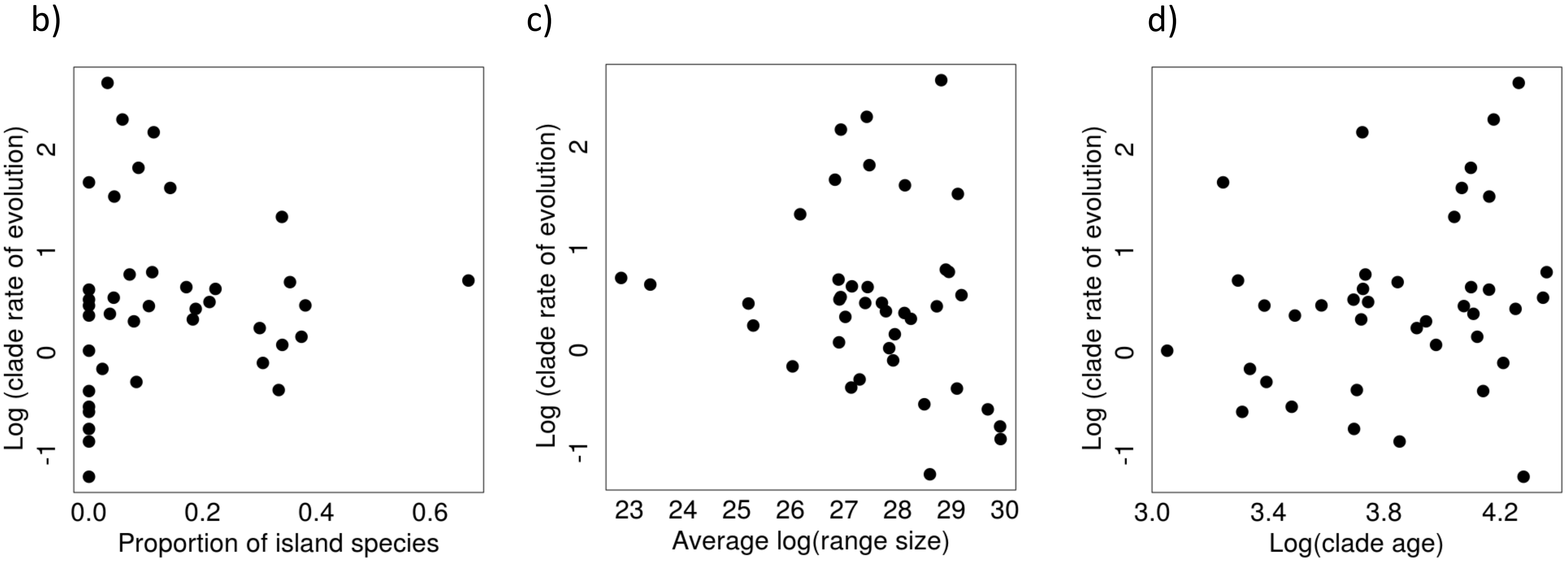

Figure S7. The relationship between clade rates of evolution and (b) proportion of island species, (c) average range size, and (d) clade age. None of the variables correlates significantly with evolutionary rates.

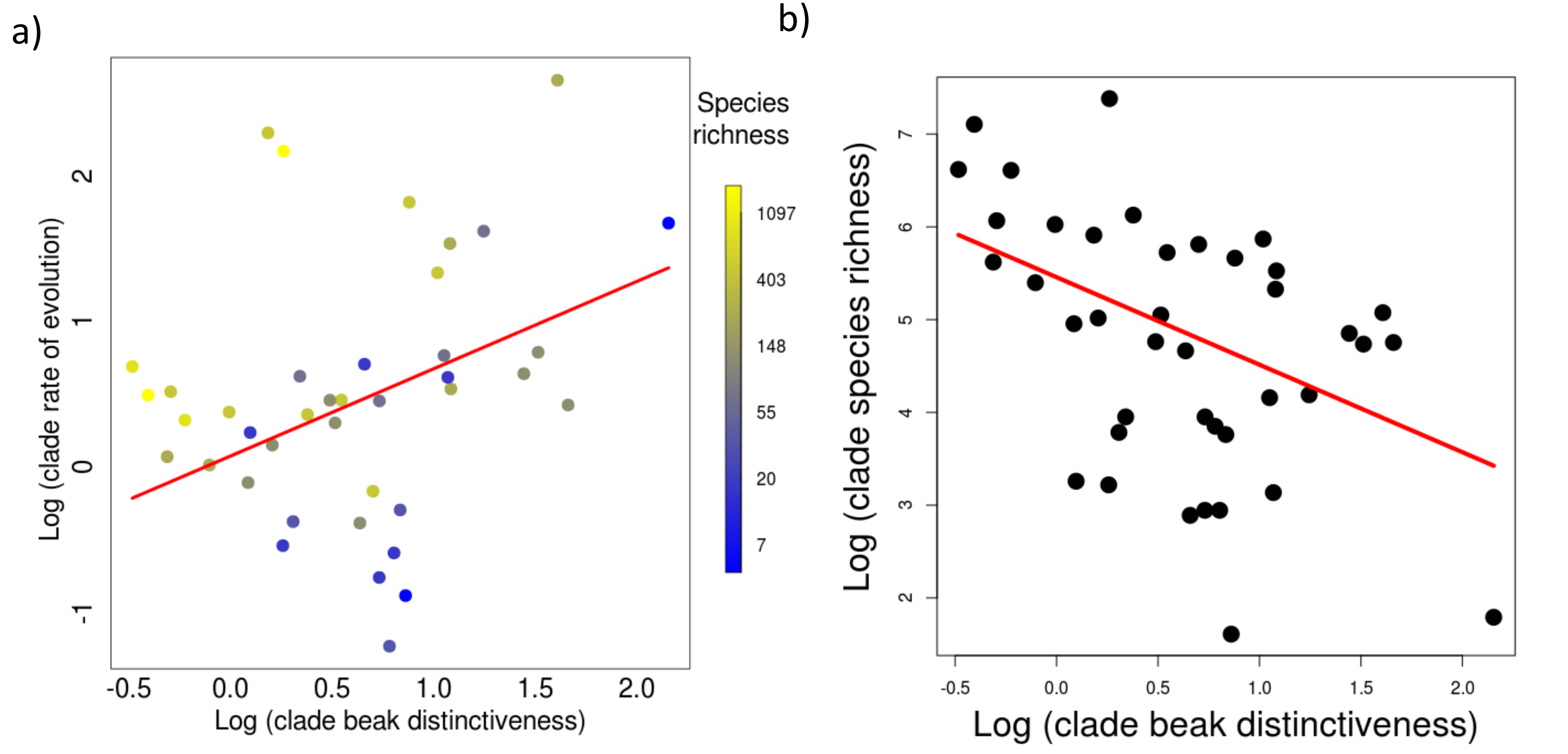

Figure S8. (a) The relationship between clade rate of evolution and clade beak distinctiveness; points are coloured by clade species richness. (b) The relationship between clade species richness and clade beak distinctiveness, p = 0.01. There is much variation to this relationship: many clades with unusual beaks have rather intermediate species richness values (e.g. *Anseriformes*, *Accipitriformes*, *Apodiformes*, *Strigiformes*), and some distinctive beak shaped clades are also species rich (e.g. *Psittacidae*).

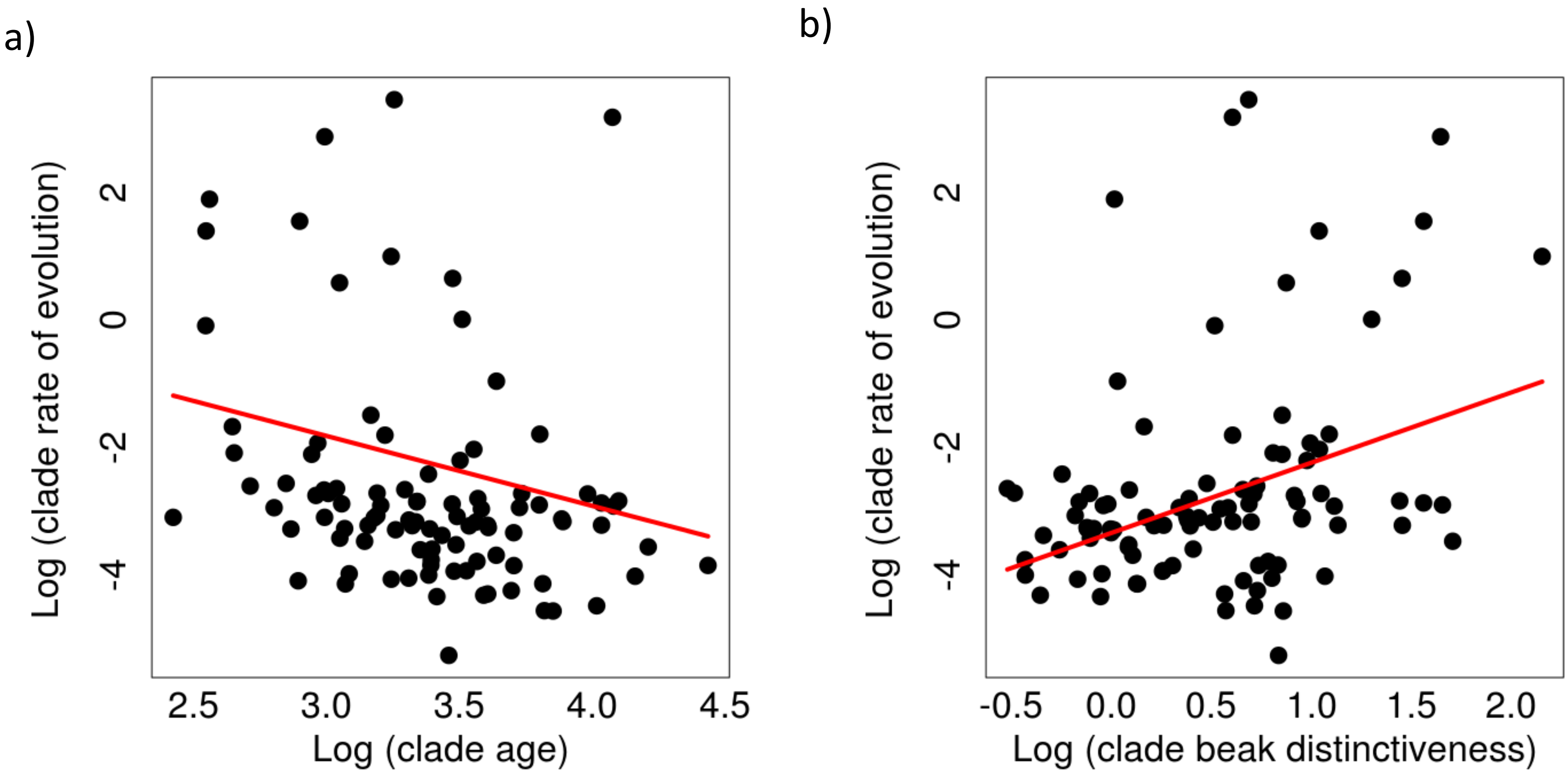

Figure S9. The relationship between clade rates of evolution and (a) clade age, $p = 0.002$, (b) clade beak distinctiveness, $p < 0.001$. A finer division of clades (especially among the Passerines) is used.

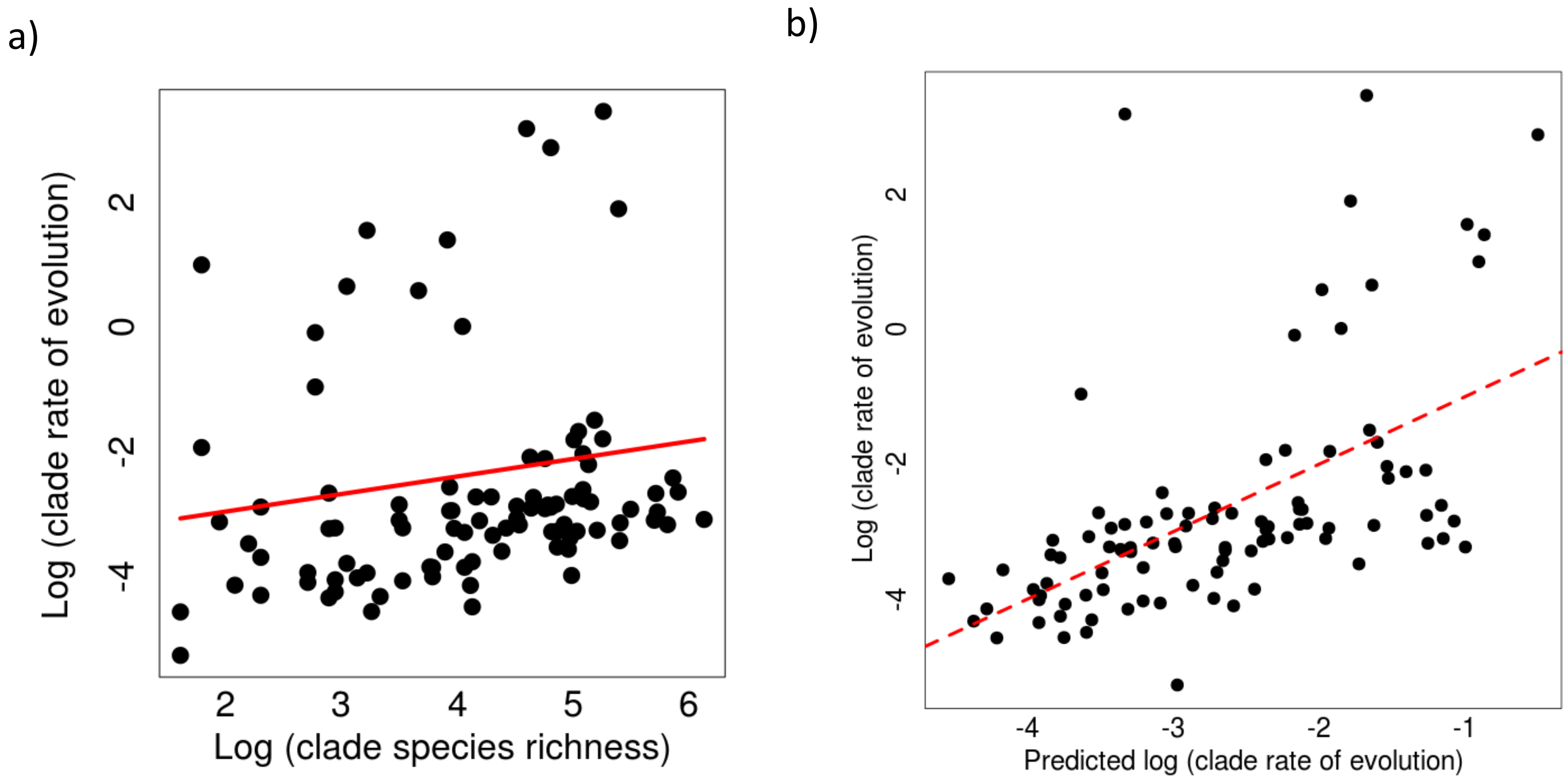

Figure S10. (a) The relationship between clade rates of evolution and (a) clade species richness, *p* = 0.014. (b) The relationship between the observed and predicted clade rate of evolution by the full PGLS model (adj. R-sq = 0.27). The dashed line indicates the 1:1 line of predicted versus observed values. A finer division of clades (especially among the Passerines) is used.

Figure S11. Multivariate rates of beak shape evolution for alternative trees built (i) using *Jetz et al 2012* and *Prum et al 2015*, (ii) using trees built including only species with genetic data (G) or the full range of species (F), (iii) using a maximum clade credibility tree with node heights set to “common ancestor heights” (CAH) or heights in the target tree (HTT). Continued overleaf.

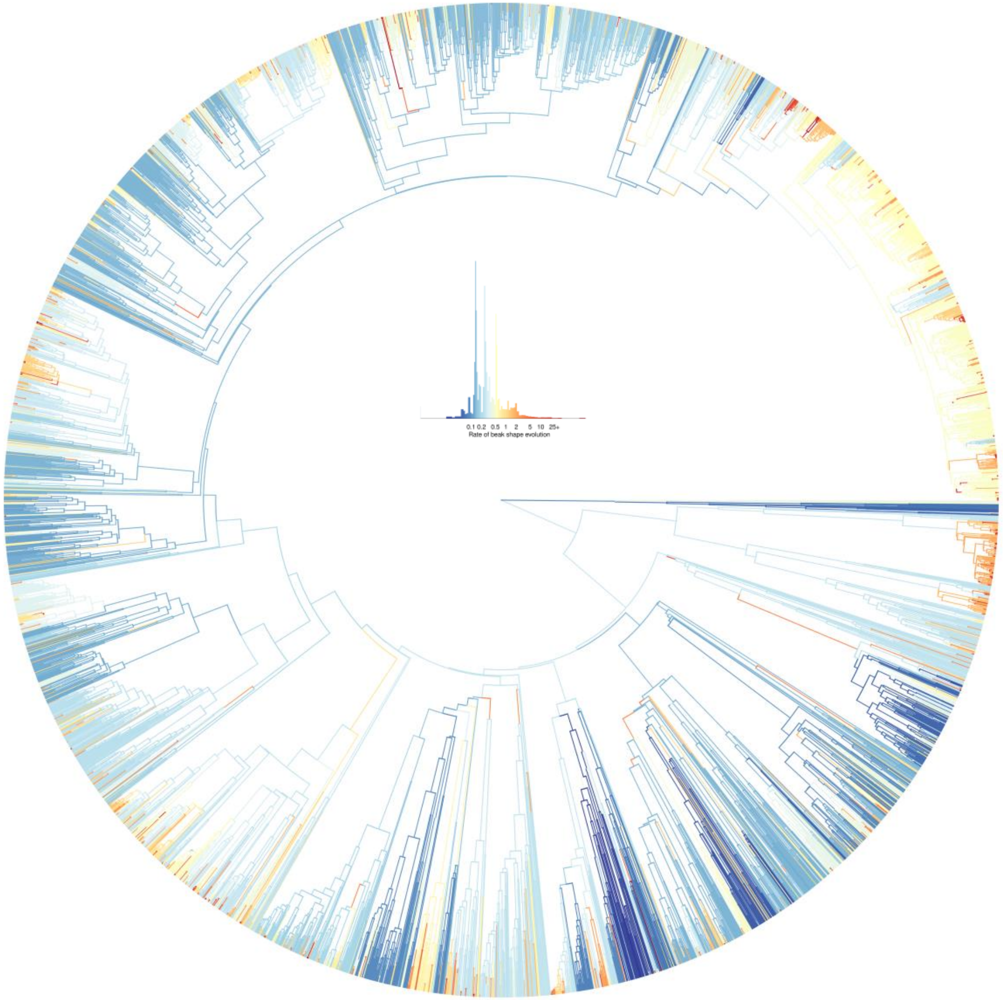

Jetz Gdata 8PCs HTT

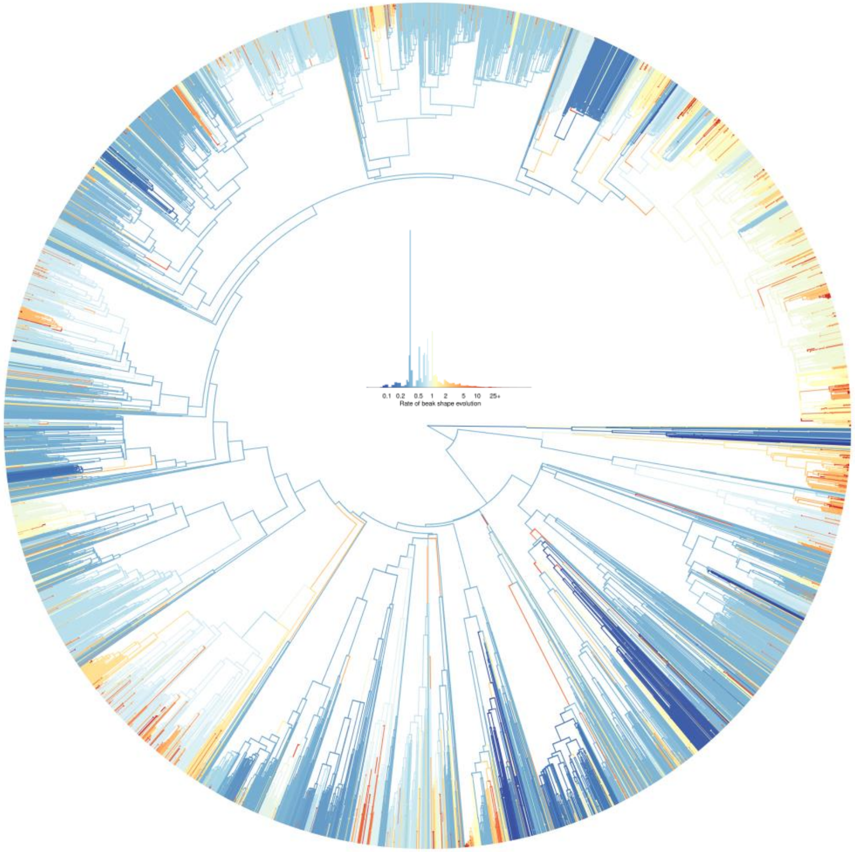

(b) Jetz Fdata 8PC CAH

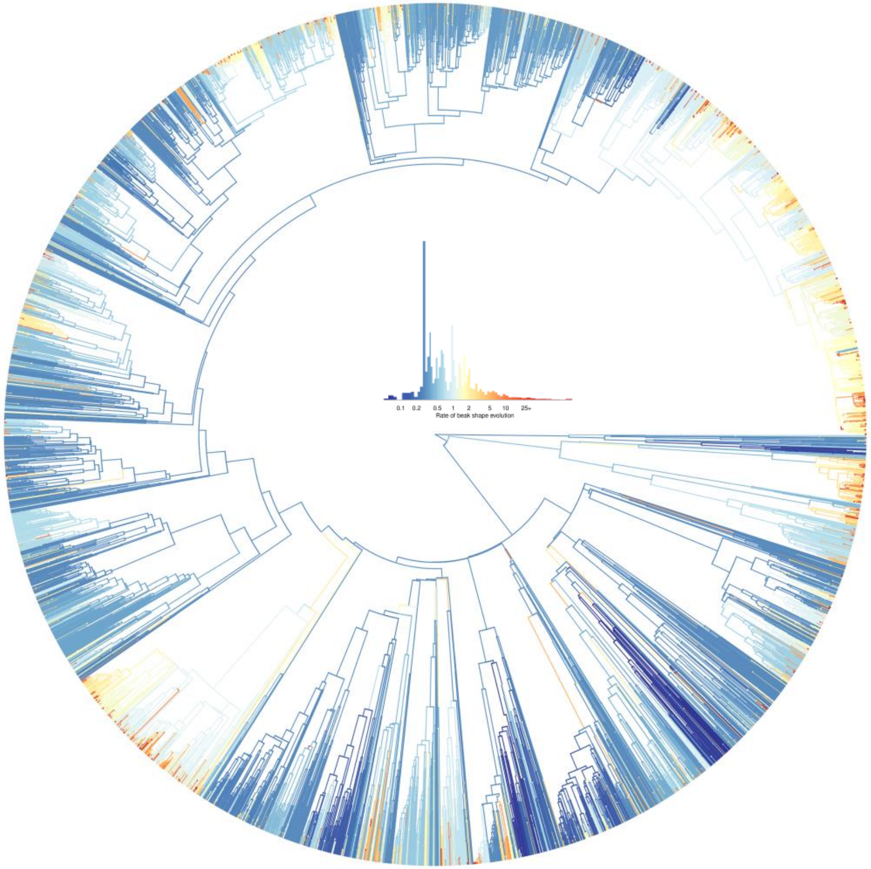

(c) Jetz Fdata 8PC HTT

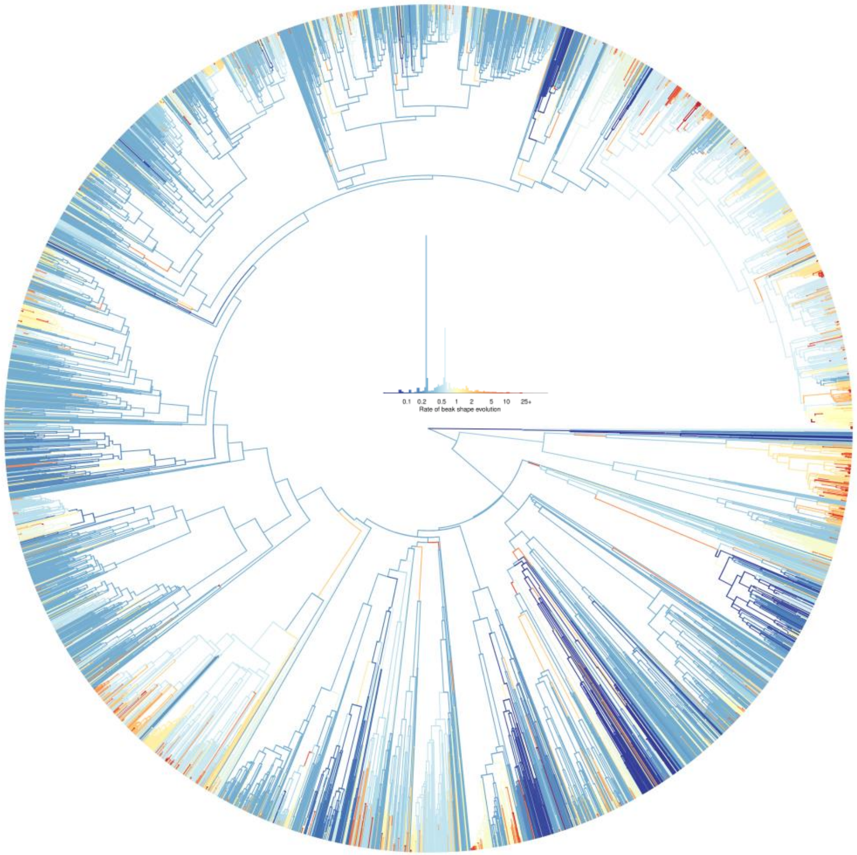

(b) Jetz Gdata 15pPC CAH

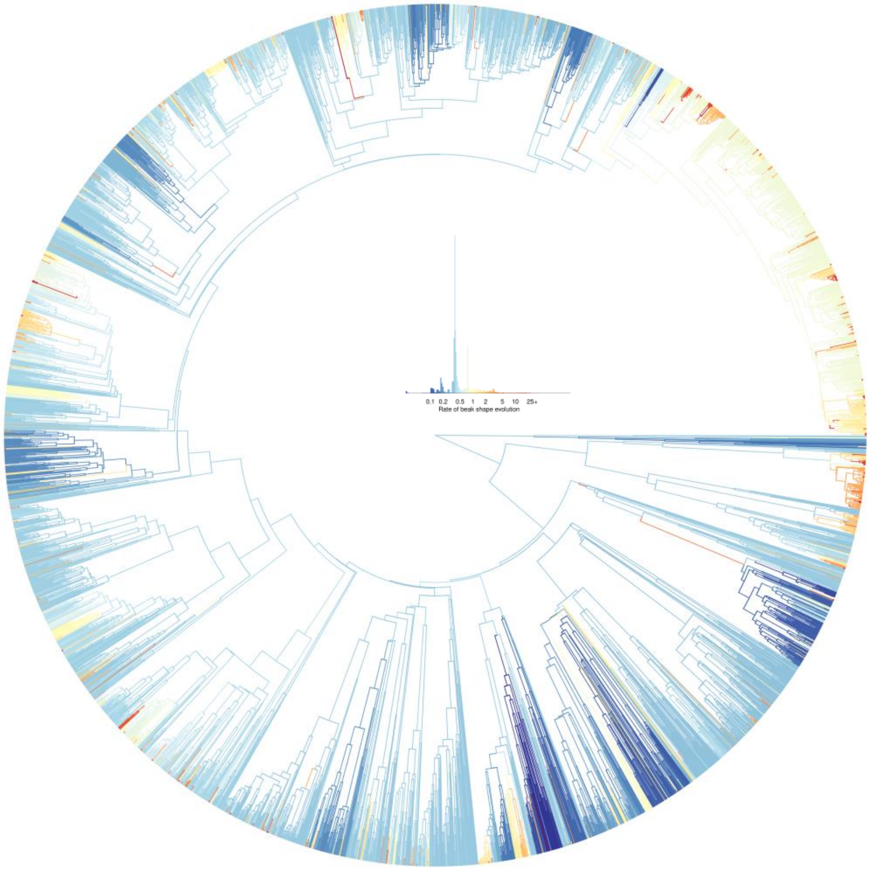

(c) Jetz Gdata 3pPC HTT

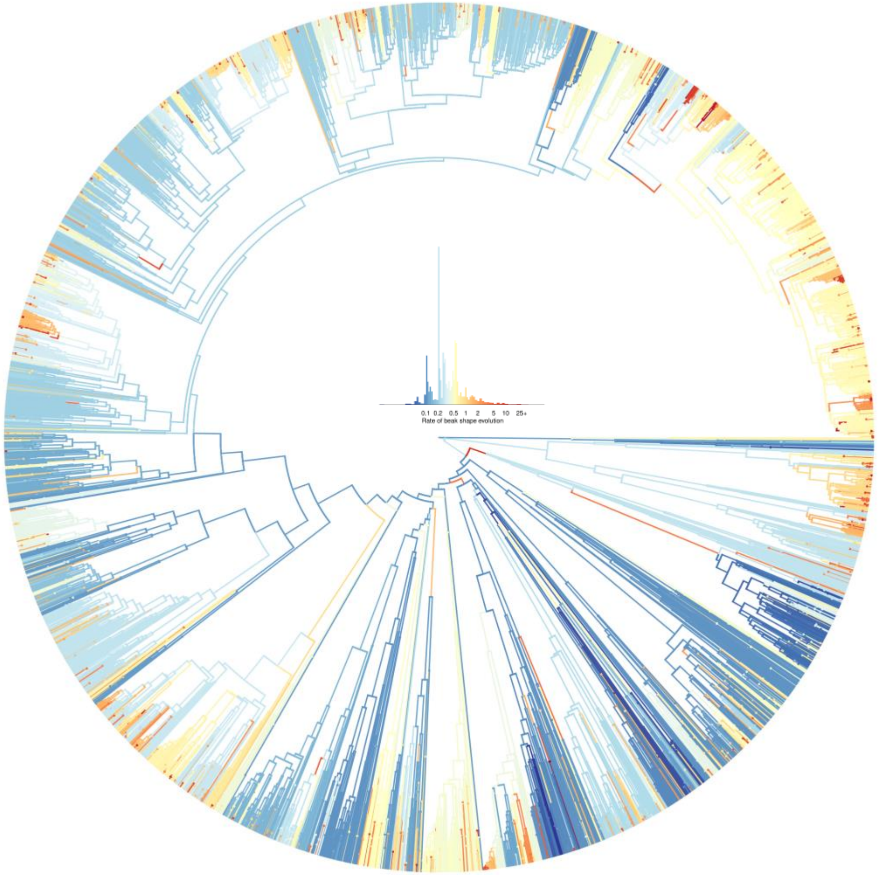

(b) Prum Gdata 8PC CAH

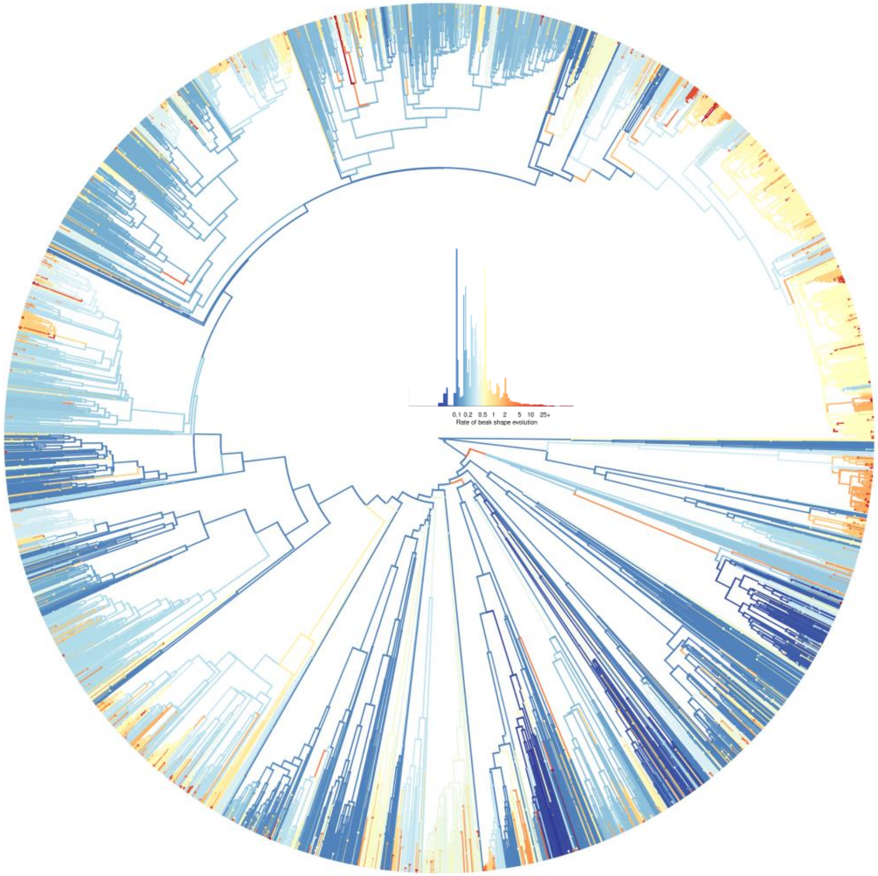

(c) Prum Gdata 8PC HTT
