# Supplementary material for: Correlates of rate heterogeneity in avian ecomorphological traits
Source: Ecol Lett. 2018 Aug 21;21(10):1505–14. doi: 10.1111/ele.13131 (PMC6175488; doi:10.1111/ele.13131)
Supplement: Supplementary file 2 [file ELE-21-1505-s002.docx]

**Table S1**. List of alternative runs used in the analyses. Trees were built using: (i) Jetz et al. (2012) and Prum et al. (2015) avian phylogenies, (ii) only species with genetic data (G) or full range of species (F), (iii) maximum clade credibility trees, setting node heights to “common ancestor heights” (CAH) or to heights in the target tree (HTT). We ran both phylogenetic (pPCA) and non-phylogenetic (PCA) principal components analysis on the data, and used a number of PC/pPC axes that covered 95% and 99% of the beak shape variation (#PCs). The models were generally run twice (#Runs), but some trees and datasets were used only once due to time constraints.

| Run | Phylogeny | Genetic vs Full data | MCC method | PCA/pPCA | # PCs | #Runs |
| --- | --- | --- | --- | --- | --- | --- |
| 1 | Jetz et al | F | CAH | PCA | 4 | 2 |
| 2 | Jetz et al | F | CAH | PCA | 8 | 2 |
| 3 | Jetz et al | F | HTT | PCA | 4 | 2 |
| 4 | Jetz et al | F | HTT | PCA | 8 | 2 |
| 5 | Jetz et al | G | CAH | PCA | 4 | 2 |
| 6 | Jetz et al | G | CAH | PCA | 8 | 2 |
| 7 | Jetz et al | G | HTT | PCA | 4 | 2 |
| 8 | Jetz et al | G | HTT | PCA | 8 | 2 |
| 9 | Prum et al | G | CAH | PCA | 4 | 1 |
| 10 | Prum et al | G | CAH | PCA | 8 | 1 |
| 11 | Prum et al | G | HTT | PCA | 4 | 1 |
| 12 | Prum et al | G | HTT | PCA | 8 | 1 |
| 13 | Jetz et al | F | CAH | pPCA | 5 | 2 |
| 14 | Jetz et al | F | CAH | pPCA | 15 | 1 |
| 15 | Jetz et al | F | HTT | pPCA | 4 | 2 |
| 16 | Jetz et al | F | HTT | pPCA | 12 | 1 |
| 17 | Jetz et al | G | CAH | pPCA | 6 | 2 |
| 18 | Jetz et al | G | CAH | pPCA | 15 | 1 |
| 19 | Jetz et al | G | HTT | pPCA | 3 | 2 |

**Table S2.** The percentage of beak shape variation described (phylogenetic) PC axes across alternative phylogenies: (i) using Jetz et al. (2012) and Prum et al. (2015), (ii) using trees built including only species with genetic data (G) or the full range of species (F), (iii) using a maximum clade credibility tree with node heights set to “common ancestor heights” (CAH) or heights in the target tree (HTT). For each run, values adding up to 99% of beak shape variation are shown.

| Run | PC1 | PC2 | PC3 | PC4 | PC5 | PC6 | PC7 | PC8 | PC9 | PC10 | PC11 | PC12 | PC13 | PC14 | PC15 |
| --- | --- | --- | --- | --- | --- | --- | --- | --- | --- | --- | --- | --- | --- | --- | --- |
| (a) PCA | | | | | | | | | | | | | | | |
| G | 0.491 | 0.258 | 0.128 | 0.050 | 0.029 | 0.020 | 0.009 | 0.004 |  | | | | | | |
| F | 0.564 | 0.295 | 0.058 | 0.031 | 0.023 | 0.011 | 0.005 | 0.004 |  | | | | | | |
| (b) phylogenetic PCA | | | | | | | | | | | | | | | |
| Jetz et al CAH G | 0.520 | 0.213 | 0.110 | 0.047 | 0.035 | 0.021 | 0.015 | 0.009 | 0.005 | 0.004 | 0.003 | 0.003 | 0.002 | 0.002 | 0.001 |
| Jetz et al HTT G | 0.665 | 0.310 | 0.014 |  | | | | | | | | | | | |
| Jetz et al CAH F | 0.579 | 0.230 | 0.058 | 0.042 | 0.029 | 0.016 | 0.010 | 0.006 | 0.005 | 0.004 | 0.003 | 0.002 | 0.002 | 0.001 | 0.001 |
| Jetz et al HTT F | 0.475 | 0.336 | 0.066 | 0.045 | 0.030 | 0.011 | 0.010 | 0.005 | 0.004 | 0.003 | 0.002 | 0.002 |  | | |

**Table S3**. Pearson’s correlation between tip rates across alternative multivariate BayesTraits runs, using a number of (phylogenetic) PC axes that explain 99% of variation in beak shape. Alternative runs are built (i) using Jetz et al. (2012) and Prum et al. (2015), (ii) using trees built including only species with genetic data (G) or the full range of species (F), (iii) using a maximum clade credibility tree with node heights set to “common ancestor heights” (CAH) or heights in the target tree (HTT). Rate values were logged to ensure a normal distribution. Unless otherwise indicated, traits represent PC axes.

| Run | correlation coefficient | t-test statistic | df | p-value |
| --- | --- | --- | --- | --- |
| Jetz et al CAH G data ~ Prum et al CAH G data | 0.978 | 298.26 | 4106 | <0.001 |
| Jetz et al HTT G data ~ Prum et al HTT G data | 0.979 | 311.12 | 4106 | <0.001 |
| Jetz et al CAH G data ~ Jetz et al HTT G data | 0.874 | 115.55 | 4106 | <0.001 |
| Jetz et al CAH G data (pPCA) ~ Jetz et al HTT G data (pPCA) | 0.642 | 53.537 | 4106 | <0.001 |
| Prum et al CAH G data ~ Prum et al HTT G data | 0.879 | 118.01 | 4106 | <0.001 |
| Jetz et al CAH F data ~ Jetz et al HTT F data | 0.754 | 85.539 | 5549 | <0.001 |
| Jetz et al CAH G data (PCA) ~ Jetz et al CAH G data (pPCA) | 0.837 | 97.862 | 4106 | <0.001 |
| Jetz et al HTT G data (PCA) ~ Jezt et al HTT G data (pPCA) | 0.843 | 102.67 | 4106 | <0.001 |

**Table S4.** Adjusted R-squared values when an interaction between clade names and individual predictors is included in the model. These analyses are run on a tree built using the Jetz et al (2012) phylogeny, including only species with genetic data, and using a maximum clade credibility tree with node heights set to “common ancestor heights”. Traits represent PC axes.

| Predictor involved in interaction | Adj R-sq |
| --- | --- |
| Log age | 0.22 |
| Log generation length | 0.21 |
| Log body mass | 0.21 |
| Mean annual temperature | 0.22 |
| Mean UVB levels | 0.22 |
| Proportion of island range | 0.22 |
| Log number of competitors | 0.22 |
| Measurement error | 0.22 |

**Table S5**. Correlates for species-specific rates of evolution. Results across alternative trees built (i) using using Jetz et al. (2012) and Prum et al. (2015), (ii) using trees built including only species with genetic data (G) or the full range of species (F), (iii) using a maximum clade credibility tree with node heights set to “common ancestor heights” (CAH) or heights in the target tree (HTT). We perform PGLS analyses only for the runs in which we used a number of PC/pPCs that covered 99% of variation in beak shape. Given the strong correlations between tip rates across alternative runs (Table S3), we only perform PGLS analyses on a subset of alternative trees and datasets. Unless otherwise indicated, traits represent PC axes.

| Predictor | Slope ± SE | *t* | *P* |
| --- | --- | --- | --- |
| Prum et al, Gdata, CAH. λ = 0.635 | | | |
| Log species’ age | -0.476 ± 0.016 | -29.753 | **<0.001***** |
| Log body mass | 0.041 ± 0.023 | 1.802 | 0.072 |
| Log generation length | 0.100 ± 0.082 | 1.223 | 0.222 |
| Mean annual temperature | 0.000 ± 0.002 | -0.104 | 0.917 |
| Mean annual UVB levels | 0.000 ± 0.000 | -2.112 | **0.035*** |
| Log range size | -0.010 ± 0.006 | -1.538 | 0.124 |
| Proportion of island range | 0.098 ± 0.047 | 2.106 | **0.035*** |
| Log number of competitors | 0.004 ± 0.013 | 0.265 | 0.791 |
| Measurement error | 1.009 ± 0.425 | 2.375 | **0.018*** |
| Jetz et al, Gdata, HTT. λ = 0.629 | | | |
| Log species’ age | -0.577 ± 0.013 | -44.511 | **<0.001***** |
| Log body mass | 0.030 ± 0.023 | 1.282 | 0.200 |
| Log generation length | 0.059 ± 0.083 | 0.713 | 0.476 |
| Mean annual temperature | 0.002 ± 0.003 | 0.772 | 0.440 |
| Mean annual UVB levels | 0.000 ± 0.000 | -2.562 | **0.011*** |
| Log range size | -0.005 ± 0.007 | -0.678 | 0.498 |
| Proportion of island range | 0.148 ± 0.049 | 2.994 | **0.003**** |
| Log number of competitors | -0.003 ± 0.014 | -0.194 | 0.847 |
| Measurement error | 1.182 ± 0.450 | 2.630 | **0.009**** |
| Jetz et al, Fdata, CAH. λ = 0.654 | | | |
| Log species’ age | -0.485 ± 0.014 | -35.924 | **<0.001***** |
| Log body mass | 0.050 ± 0.018 | 2.700 | **0.007**** |
| Log generation length | 0.078 ± 0.066 | 1.177 | 0.239 |
| Mean annual temperature | -0.002 ± 0.002 | -1.141 | 0.254 |
| Mean annual UVB levels | 0.000 ± 0.000 | -1.794 | 0.073 |
| Log range size | -0.001 ± 0.005 | -0.293 | 0.770 |
| Proportion of island range | 0.078 ± 0.035 | 2.224 | **0.026*** |
| Log number of competitors | -0.008 ± 0.010 | -0.777 | 0.437 |
| Measurement error | 0.648 ± 0.337 | 1.923 | 0.055 |
| Jetz et al, Gdata, CAH, p(PCA) analysis. λ = 0.502 | | | |
| Log species’ age | -0.553 ± 0.016 | -34.911 | **<0.001***** |
| Log body mass | 0.037 ± 0.022 | 1.725 | 0.085 |
| Log generation length | 0.094 ± 0.077 | 1.228 | 0.219 |
| Mean annual temperature | -0.006 ± 0.002 | -2.476 | **0.013*** |
| Mean annual UVB levels | 0.000 ± 0.000 | -0.899 | 0.369 |
| Log range size | -0.008 ± 0.007 | -1.222 | 0.222 |
| Proportion of island range | 0.170 ± 0.047 | 3.620 | **<0.001***** |
| Log number of competitors | -0.009 ± 0.013 | -0.684 | 0.494 |
| Measurement error | 1.351 ± 0.435 | 3.102 | **0.002**** |

**Table S6**. Correlates for clade rates of evolution; results across alternative trees built (i) using using Jetz et al Jetz et al. 2012 and Prum et al Prum et al. 2015, (ii) using trees built including only species with genetic data (G) or the full range of species (F), (iii) using a maximum clade credibility tree with node heights set to “common ancestor heights” (CAH) or heights in the target tree (HTT). We perform PGLS analyses only for the runs in which we used a number of PC/pPCs that covered 99% of variation in beak shape. Given the strong correlations between evolutionary rates across alternative runs (Table S3), we only perform PGLS analyses on a subset of alternative trees and datasets. Unless otherwise indicated, traits represent PC axes.

| Predictor | Slope ± SE | *t* | *P* |
| --- | --- | --- | --- |
| Prum et al, Gdata, CAH, adj. R-squared = 0.51 | | | |
| Log clade age | -0.157 ± 0.270 | 0.800 | 0.430 |
| Log clade beak distinctiveness | 1.012 ± 0.222 | 4.556 | **<0.001***** |
| Log clade species richness | 0.564 ± 0.112 | 4.556 | **<0.001***** |
| Log average range size | -0.142 ± 0.081 | -1.753 | 0.089 |
| Proportion of island species | -0.183 ± 0.839 | -0.218 | 0.829 |
| Log average number of competitors | -0.373 ± 0.174 | -2.137 | **0.040*** |
| Jetz et al, Gdata, HTT, adj. R-squared = 0.16 | | | |
| Log clade age | 0.170 ± 0.640 | 0.265 | 0.792 |
| Log clade beak distinctiveness | 0.568 ± 0.424 | 1.342 | 0.189 |
| Log clade species richness | 0.634 ± 0.252 | 2.515 | **0.017*** |
| Log average range size | -0.193 ± 0.169 | -1.143 | 0.261 |
| Proportion of island species | -0.814 ± 1.718 | -0.474 | 0.639 |
| Log average number of competitors | -0.223 ± 0.349 | -0.640 | 0.527 |
| Jetz et al, Fdata, CAH, adj. R-squared = 0.22 | | | |
| Log clade age | -0.471 ± 0.414 | -1.138 | 0.263 |
| Log clade beak distinctiveness | 0.789 ± 0.240 | 3.285 | **0.002**** |
| Log clade species richness | 0.376 ± 0.138 | 2.727 | **0.010*** |
| Log average range size | -0.045 ± 0.091 | -0.496 | 0.623 |
| Proportion of island species | 0.096 ± 0.767 | 0.125 | 0.901 |
| Log average number of competitors | -0.160 ± 0.204 | -0.786 | 0.438 |
| Jetz et al, Gdata, CAH, phylogenetic PCA analysis, adj. R-squared = 0.47 | | | |
| Log clade age | -0.069 ± 0.380 | -0.183 | 0.856 |
| Log clade beak distinctiveness | 1.198 ± 0.265 | 4.527 | **<0.001***** |
| Log clade species richness | 0.518 ± 0.129 | 4.030 | **<0.001***** |
| Log average range size | -0.153 ± 0.089 | -1.727 | 0.094 |
| Proportion of island species | -0.079 ± 0.904 | -0.088 | 0.931 |
| Log average number of competitors | -0.403 ± 0.196 | -2.054 | **0.048*** |

**Table S7**. Correlates for clade rates of evolution; d.f. = 6,90; adjusted R-squared = 0.27. A finer division of clades (especially among the Passerines) is used. These analyses are run on a tree built using the Jetz et al (2012) phylogeny, including only species with genetic data, and using a maximum clade credibility tree with node heights set to “common ancestor heights”. Traits represent PC axes.

| Predictor | Slope ± SE | *t* | *P* |
| --- | --- | --- | --- |
| Log clade age | -1.205 ± 0.387 | -3.115 | **0.002**** |
| Log clade beak distinctiveness | 1.536 ± 0.318 | 4.826 | **<0.001***** |
| Log clade species richness | 0.377 ± 0.150 | 2.508 | **0.014*** |
| Log average range size | -0.189 ± 0.140 | -1.346 | 0.182 |
| Proportion of island species | -0.070 ± 0.885 | -0.080 | 0.937 |
| Log average number of competitors | 0.108 ± 0.197 | 0.549 | 0.585 |

**Table S8**. Correlates for clade rates of evolution; d.f. = 5,34; adjusted R-squared = 0.17. Species richness is excluded as a predictor. These analyses are run on a tree built using the Jetz et al (2012) phylogeny, including only species with genetic data, and using a maximum clade credibility tree with node heights set to “common ancestor heights”. Traits represent PC axes.

| Predictor | Slope ± SE | *t* | *P* |
| --- | --- | --- | --- |
| Log clade age | 0.559 ± 0.384 | 1.456 | 0.155 |
| Log clade beak distinctiveness | 0.610 ± 0.266 | 2.291 | **0.028*** |
| Log average range size | -0.200 ± 0.099 | -2.026 | 0.051 |
| Proportion of island species | -0.409 ± 1.028 | -0.398 | 0.693 |
| Log average number of competitors | 0.091 ± 0.172 | 0.528 | 0.601 |

**REFERENCES**

Jetz, W., Thomas, G.H., Joy, J.B., Hartmann, K. & Mooers, A.O. (2012). The global diversity of birds in space and time. *Nature*, 491, 444-448.

Prum, R.O., Berv, J.S., Dornburg, A., Field, D.J., Townsend, J.P., Lemmon, E.M. *et al.* (2015). A comprehensive phylogeny of birds (Aves) using targeted next-generation DNA sequencing. *Nature*, 526, 569-573.
